# RefXAS: an open access database of X-ray absorption spectra

**DOI:** 10.1107/S1600577524006751

**Published:** 2024-08-27

**Authors:** Sebastian Paripsa, Abhijeet Gaur, Frank Förste, Dmitry E. Doronkin, Wolfgang Malzer, Christopher Schlesiger, Birgit Kanngießer, Edmund Welter, Jan-Dierk Grunwaldt, Dirk Lützenkirchen-Hecht

**Affiliations:** a Fk. 4, Physik Bergische Universität Wuppertal Gaußstraße 20 D-42097Wuppertal Germany; b Institute for Chemical Technology and Polymer Chemistry Karlsruhe Institute of Technology Engesserstraße 20 D-76131Karlsruhe Germany; c Technische Universität Berlin Hardenbergstraße 36 D-10623Berlin Germany; d Institute of Catalysis Research and Technology Karlsruhe Institute of Technology Hermann-von-Helmholtz-Platz 1 D-76344Eggenstein-Leopoldshafen Germany; e Deutsches Elektronen-Synchrotron (DESY) Notkestraße 85 D-22607Hamburg Germany; ESRF – The European Synchrotron, France

**Keywords:** X-ray absorption fine structure, metadata, reference database, quality control, data format

## Abstract

Under DAPHNE4NFDI, the X-ray absorption spectroscopy (XAS) reference database, RefXAS, has been set up. For this purpose, we developed a method to enable users to submit a raw dataset, with its associated metadata, via a dedicated website for inclusion in the database. Implementation of the database includes an upload of metadata to the scientific catalogue and an upload of files via object storage, with automated query capabilities through a web server and visualization of the data and files. Based on the mode of measurements, quality criteria have been formulated for the automated check of any uploaded data. In the present work, the significant metadata fields for reusability, as well as reproducibility of results (FAIR data principles), are discussed. Quality criteria for the data uploaded to the database have been formulated and assessed. Moreover, the usability and interoperability of available XAS data/file formats have been explored. The first version of the RefXAS database prototype is presented, which features a human verification procedure, currently being tested with a new user interface designed specifically for curators; a user-friendly landing page; a full list of datasets; advanced search capabilities; a streamlined upload process; and, finally, a server-side automatic authentication and (meta-) data storage via MongoDB, PostgreSQL and (data-) files via relevant APIs.

## Abstract 2

The RefXAS database under DAPHNE4NFDI enables users to access quality-controlled, curated X-ray absorption spectra of references along with important metadata and to share their data with the research community in easy steps.

## 1. Introduction

Implementation of research data management along with high-level/rapid data analysis is the common challenge faced by photon and neutron science communities, encompassing users from a broad range of disciplines. The DAPHNE4NFDI consortium (Barty, 2023 ▸) serves a broad community of researchers, employing a wide range of photon and neutron techniques. One of the important synchrotron based techniques is X-ray absorption spectroscopy (XAS), which is employed to analyse solid materials, in particular amorphous materials, disordered or multicomponent materials (George & Pickering, 2013 ▸; Bertagnolli, 1989 ▸; Calvin, 2013 ▸). Due to its vast application in diverse fields, XAS has become an essential tool for studying catalytic reactions, battery materials, geological and biological samples, cultural heritage objects *etc.* (Lamberti & van Bokhoven, 2016 ▸; Timoshenko & Roldan Cuenya, 2021 ▸). Evaluation of XAS data often involves comparison with experimental or theoretical calculated reference spectra (Gaur & Shrivastava, 2015 ▸; Wu *et al.*, 2022 ▸), hence the quality of these spectra and documentation of the metadata is critical for any user.

Data analysis includes not only the reduction of the datasets by, for example, pre- and post-edge background subtraction, normalization, and curve fitting (Calvin, 2013 ▸), but also a comparison with reference spectra of well defined reference materials (Calvin, 2013 ▸), a principal component analysis and a linear combination fit with suitable reference materials (*e.g.* Wasserman *et al.*, 1999 ▸; Ressler *et al.*, 1999 ▸; Isaure *et al.*, 2002 ▸).

This is particularly true if *in situ* data are measured during a chemical reaction (catalysis, electrochemistry *etc.*) or during variation of pressure, temperature and other constraints in a time-resolved manner (Timoshenko & Roldan Cuenya, 2021 ▸; Doronkin *et al.*, 2020 ▸). Nowadays, the successful developments of the time-dependent EXAFS technique such as Quick-EXAFS (Frahm, 1989 ▸; Stötzel *et al.*, 2010 ▸; Frenkel *et al.*, 2013 ▸; Müller *et al.*, 2015 ▸) lead to increasingly large amounts of data that can be challenging to analyse. To address these issues, there is a need for a well curated database that can help to manage and analyse the data efficiently and reliably (Asakura *et al.*, 2018 ▸).

Beginning with the early days of XAS in the late 1970s, large amounts of reference data have been measured and stored, and several databases for XAS spectra have appeared, for example, see the review by Asakura *et al.* (2018 ▸). As an example, the Farrel Lytle database (Boyanov & Segre, 1995 ▸) was a widely used database for XAS data in the past, containing information on a range of materials, and has become an essential tool for researchers and practitioners in the field of XAS. Though there are shortcomings such as data lacking information of the sample preparation and the experimental conditions, still it has been one of the nice initiatives for XAS databases with the limited resources available. The old data formats are used for many of the datasets and users need to have some prior knowledge of the experimental mode before using those datasets. There is no option for data upload by any general user.

The XASLIB database (https://xaslib.xrayabsorption.org) is another good example of an XAS database. The elements are arranged nicely in the form of a periodic table and there are about 277 spectra available from 20 elements. For uploading the data, user needs to create a login at the database and then data can be uploaded only in ASCII format along with some metadata fields (*e.g.* sample name, absorbing element, edge, monochromator *d*-spacing *etc*.). It also has some limitations, such as being based on user submissions and lacking comprehensive information, in particular on the details of the experimental conditions during the measurements (*i.e.* metadata on the setups and sample are not deployed).

The Materials Data Repository (MDR) XAFS database (Ishii *et al.*, 2023 ▸) has been constructed by integrating XAFS databases in Japan and contains around 2174 spectra ranging from soft to hard X-ray energies. For cross-searchability from different metadata reported, sample nomenclature has been used in this database so that the differences in the local metadata provided by the facilities do not affect the search. The database developers have compiled the energy calibration policies of participating institutions for comparing the spectra. One of the shortcomings is that data quality is not included in the criteria for this database and hence the use of data is at the discretion of the user, which can limit the use of the database.

Another useful initiative from the MDR XAFS DB group is the XAFS DB portal (https://materiage.org/xafs/). This portal has been developed for cross-searching XAFS spectral data on a worldwide scale. Currently, XASLIB and MDR XAFS DB are part of this portal.

Regarding XAS databases from Japan, one more XAFS Standard Sample Database has been made available by beamline BL4B2 at SPring-8 (Ofuchi *et al.*, 2024 ▸). The database has a collection of XAS data of standard samples measured on the beamline. In order to control the quality of the data, only data measured by corresponding beamline staff using the same procedure have been included and not those measured by users.

The SSHADE/FAME database (Kieffer & Testemale, 2016 ▸) created by the French X-ray spectroscopy beamlines at the ESRF also provide XAS spectra linked to a detailed sample description and validated quality. The database consists of spectra of standards and characteristic samples provided by the beamlines users. At the ESRF, beamline ID21 also hosts a database of S *K*-edge XANES spectra of sulfur reference compounds and P *K*-edge XANES spectra of phospho­rous reference compounds acquired by the users (see https://www.esrf.fr/UsersAndScience/Experiments/XNP/ID21).

The Diamond Light Source XAS data repository has also been reported (Cibin *et al.*, 2020 ▸) which contains XAS data of reference sample spectra measured at beamline B18. Furthermore, among others AcReDaS (Rossberg *et al.*, 2014 ▸) is a database for X-ray spectroscopic data of actinides and other samples which is only available for registered users. For the submitted data, some metadata as well as preparation procedures are required; however, clear and objective quality criteria are not implemented.

In addition to the above-mentioned databases consisting of experimental measured spectra, the collection of computed XAS spectra are also made available under the Materials Project (Jain *et al.*, 2013 ▸), which includes *K*-edge XANES spectra (Mathew *et al.*, 2018 ▸) and *L*-edge spectra (Chen *et al.*, 2021 ▸) for unique materials generated using a high-throughput computational workflow employing *FEFF9* code (Rehr *et al.*, 2010 ▸). This project enables users to compute the properties of all inorganic materials and also provide the data and associated analysis algorithms.

Regarding data collection, data analysis methods, the statistical treatment of XAFS data as well as reporting of XAFS data, one of the first reports was published by Sayers (2000*a* ▸). After a gap of almost a decade, XAS data format standardization has been addressed again in the XAS community (Ravel *et al.*, 2012 ▸) during the Q2XAFS workshop in 2011 (Ascone *et al.*, 2012 ▸). In this report, several challenges in making the standards for data formatting to facilitate sharing of data were discussed in detail. The aim of these Q2XAFS (International Workshop on Improving Data Quality and Quantity for XAFS Experiments) workshops was to establish new standards and criteria for XAFS experiments and analyses, as well as to discuss new data formats, databases and ideas for data deposition. XAS Data Interchange (XDI) format (Ravel & Newville, 2016 ▸) has been suggested for exchange of a single XAS spectrum and Hierarchical File System version 5 (HDF5) format for multispectral X-ray experiments. Next, Q2XAFS 2017 was held at Diamond Light Source (UK) where the first agreed data formats and standards for XAFS data and deposition were discussed. Following the outcome of this workshop, it has been suggested to study across many XAFS beamlines and facilities and hence perform a round robin study on well defined samples (Chantler *et al.*, 2018 ▸). In this series, the recent developments on the XAS data formats and quality have been presented during the Q2XAFS workshop held at the Australian Synchrotron, ANSTO, Melbourne (Q2XAFS, 2023 ▸). In this context, an application definition for XAS (processed data) based on NeXus (NeXus, 2024 ▸) has been under discussion within the XAS community. NeXus is an attempt to define dictionaries of metadata (mostly for HDF5) for data from X-ray, neutron and muon facilities. NeXus describes the structure of a hierarchical data format that allows representation of data and metadata in a tree-like form (HDF5).

Recently, Meyer *et al.* (2024 ▸) published recommendations to standardize reporting, execution and interpretation of XAS measurements. They recommended to include detailed information on sample preparation, XAS measurements at the facility used, XAS data analysis and the *in situ* process involved.

To summarize, there are a number of databases available to the XAS community. Some of these are made available from specific beamline measurements which are useful but lack comprehensive metadata information for reuse of the data. Some databases are dedicated to measurements at a particular elemental edge, which are useful for the dedicated user community of the beamline. The quality criteria for the inclusion of data are not discussed in most of the cases and there is no interface developed for visualizing/pre-processing the actual data on the database website.

This paper presents the RefXAS reference database, which is specifically designed for managing, visualizing, storing and pre-processing XAS data. The project aims to establish a comprehensive XANES & EXAFS database for functional materials, with a focus on raw and processed data, and a user-friendly interface for data submission and quality assessment. The database will include real spectra and metadata, and will be well curated, benefiting both contributing researchers and users. After successfully developing the interface and uploading metal foil references in the initial phase, the project will target functional materials (*e.g.* catalysts, photovoltaic, piezoelectric, thermoelectric and magnetic materials) with the further aim to support a wide range of research areas like biology, geology or cultural heritage. The objective is to build a self-accelerating database with the support of the XAS user community and transfer the knowledge and experience gained to other areas. The project seeks to make a significant contribution to XAS research and materials development, and establish standards for XAS data management and analysis applicable to other fields. Its user-friendly interface and open access (with login) will make it easily accessible to researchers with various technical backgrounds.

Developing a reference database for XAS requires careful planning, processing and a solid understanding of the underlying physics and techniques. The first step taken is to define the metadata fields that can describe XAS experiments and documenting this information along with the data making the measured data reusable by any researcher. Another important aspect is that users of the database should also be able to estimate the quality of each dataset by looking at the formulated quality criteria. Given the diversity of available samples, it was planned to start with important references, *i.e.* metal foils, since those foils are stable, easy to handle and well reproducible. Also, sample preparation has no substantial influence on the measurements, and allows accurate comparison of data from different beamlines as well as from laboratory setups. We will also discuss plans to expand the database with spectra from other sample types in the future, *i.e.* powder materials (oxides, nitrides, ceramics, geo-materials) as well as liquids.

## 2. Database structure

For the present database, we have categorized metadata fields under ‘Sample’, ‘Spectra’, ‘Instrument’ and ‘Bibliography’, and further sub-fields were defined under these categories. Hence, the metadata fields include contributions from users as well as experimental facilities.

### 2.1. Metadata fields

The defined (meta)data fields for an uploaded XAS spectrum are given in Table 1 ▸. These fields are formulated to provide concise information (Gaur *et al.*, 2023 ▸) about the sample, bibliography, spectra and instrument used to acquire the XAS spectra.

Regarding the sample, the user needs to uniquely identify the samples and the corresponding processing so that they can be tracked through logbooks and datasets. The identifier should be unique and persistent even though the samples themselves may not always be persistent. The sample composition as well as the phase/structure would not be the same during the different steps. However, the sample IDs for the different steps should be related to each other. A specific case for a sample during different stages of the life cycle of a catalyst is shown in Fig. S1 of the supporting information.

In this regard, IGSN (International Generic Sample Number) IDs provided by the IGSN organization (Lehnert *et al.*, 2021 ▸) and registered through DataCite services (https://support.datacite.org/docs/about-igsn-ids-for-material-samples) create an efficient way to manage research samples, making it easier for researchers to keep track of them and ensure they can be located when needed.

In conclusion, each metadata field holds significant value and explains the spectra present in the database. By providing a comprehensive set of metadata fields, the database can facilitate the interpretation of XAS spectra and help to advance them in the field of XAS. It can also be connected to electronic laboratory notebooks or sample databases [*e.g.* LabIMotion (Dolcet *et al.*, 2023 ▸), eLabFTW (https://www.elabftw.net/) *etc*.].

Additionally, we defined and included metadata fields specifically designed to emphasize information on each manually added beamline (see Table S1 of the supporting information for details). Initially, we defined a metadata schema for a synchrotron source and will follow up with relevant fields for a laboratory source.

### 2.2. Data/metadata formats – interoperability

**Figure 1:**
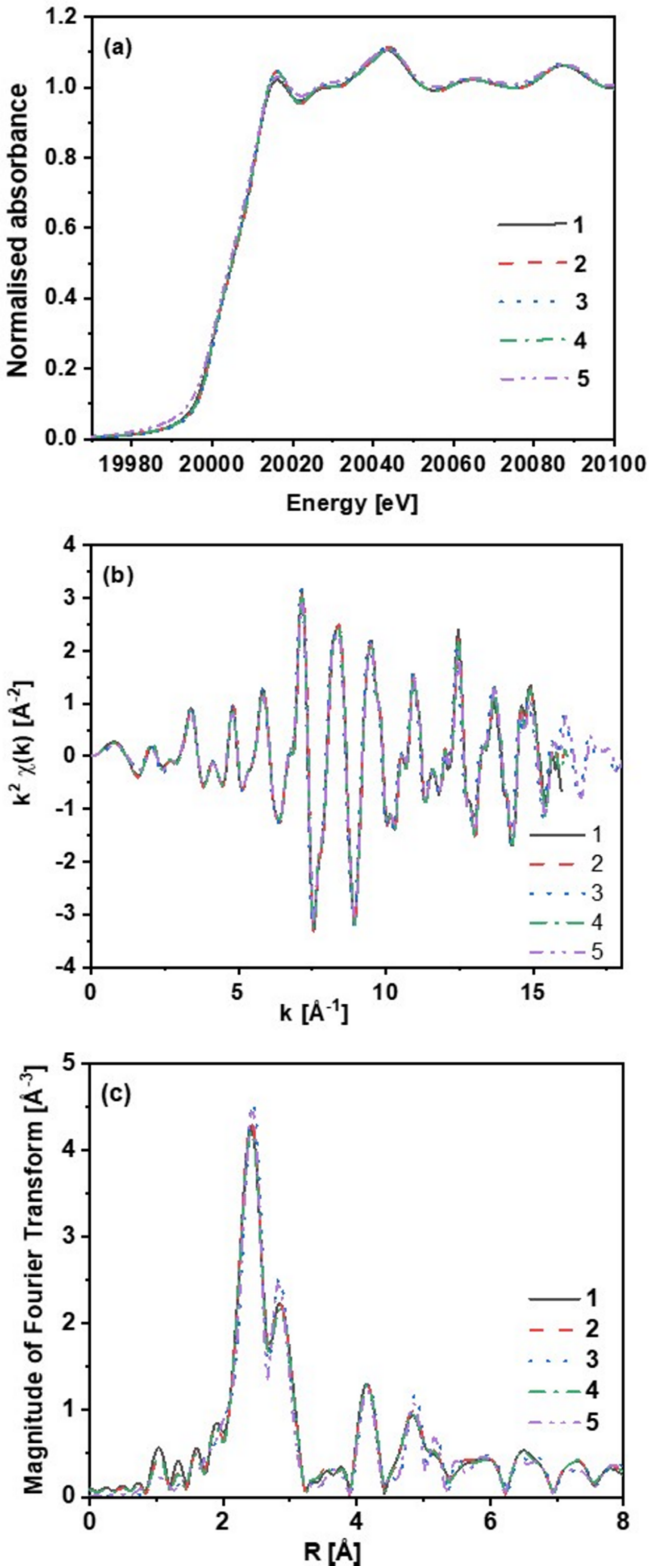
Comparison of Mo foil measurements at the *K*-edge energy (20 000 eV) obtained from different synchrotron facilities. (*a*) Normalized absorbance. (*b*) *k*^2^-weighted EXAFS fine structure *k*^2^χ(*k*) treated with identical background-subtraction procedures. (*c*) Magnitude of Fourier transformed spectra.

One of the applications of such a curated database is that it would be possible to compare the data for identical samples from different facilities and hence the effect of different parameters of an instrument on the data quality can be studied. As an example, Fig. 1 ▸ shows the comparison of Mo foils measured at XAS beamlines from different synchrotron facilities. As illustrated in Fig. 1 ▸, we present an example of Mo foil spectra at the *K*-edge from five different beamlines. Data analysis was performed using the software package *IFEFFIT* which includes *Athena* and *Artemis* (Ravel & Newville, 2005 ▸). The pre-processing steps involved calibration to the theoretical reported edge energy using the first maximum in the derivative spectrum, subtraction of a smooth background from the μ(*E*) data, and normalization by fitting a pre- and post-edge line for the determination of the edge step (Gaur *et al.,*, 2013 ▸).

However, Mo foils may not be identical (*e.g.* thickness, purity *etc.*) and thus their data are also affected by these factors other than beamline parameters. Hence, identical samples need to be measured at different XAS facilities (synchrotron/laboratory) using either their own or standardized analytical protocols. This is the basic idea behind the round robin test (Chantler *et al.*, 2018 ▸) which could further help to standardize the data/metadata fields across different laboratories. Note that Kelly *et al.* (2009 ▸) performed a similar exhaustive study where they compared the Cu and Pd *K*-edge EXAFS spectra measured at different beamlines at synchrotron facilities across the world.

These pre-processing steps were performed using *Athena*. Fitting of the EXAFS data in *k*-space as well as *R*-space was done using *Artemis* by generating a theoretical model from available crystallographic data of Mo. The first two paths (*i.e.* Mo–Mo1 and Mo–Mo2) were fitted to the experimental data in *R*-space (fit range 1.0–3.2 Å) to determine the energy shifts (Δ*E*_0_) and structural parameters, including changes in the path length (Δ*R*), passive electron reduction factor (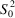), coordination number (*N*) and relative mean-square displacement of the atoms (Debye–Waller factor, σ^2^). For the two paths, 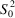 and Δ*E*_0_ were kept the same, but separate *R* and σ^2^ parameters were defined. The value of *N* is fixed to its crystallographic values for the two paths. These details about the pre-processing and analysis are important for making any comparison of the results obtained from XAS data.

Fitting results given in Table 2 ▸ showed good agreement between the bond lengths and Debye–Waller factors for Mo–Mo1/Mo2 obtained from XAS data measured at different beamlines. The 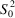 values found to vary from 0.94 to 1.03 were also comparable within the obtained error bars. Hence, in the case of Mo foils measured at different beamlines, the results obtained show that the data quality is comparable. As part of our efforts to establish a reliable reference database for XAS, we have carefully devised a series of initial steps. These steps aim to address the essential aspects of sample selection, preparation and data quality, laying a strong foundation for the development of a comprehensive and robust database.

Initially, metal foil spectra will be uploaded and tested as they exhibit appropriate thickness and homogeneity, ensuring high-quality spectral data. They are stable and easy to handle, which promotes smooth experimental procedures and minimizes sample-related issues. Metal foils are widely available, allowing researchers to access and use these reference samples across various laboratories and beamlines. Their preparation has minimal influence on the acquired data, ensuring accurate representation of the material properties rather than preparation artefacts. On comparing data obtained from different beamlines, distinct features become evident. After uploading and testing metal foil spectra based on the defined quality criteria in the initial phase, we plan to upload the spectra of functional materials. Starting with the metal oxides, nitrides, carbides *etc.* more complex sample spectra will be uploaded. Note that in the case of each sample spectrum, the spectrum of the reference foil or compound measured simultaneously should be uploaded. This has been included in the ‘Spectra’ section along with the other metadata fields (Table 1 ▸), so that each sample entry of the database includes the measurement of elemental foil or available reference. This reference spectrum will be helpful in checking the calibration procedure employed and also to retrieve information about beamline parameters, measurement statistics *etc.*

### 2.3. Automated data processing

Before measured data are submitted to the expert curator and finally uploaded into the database, automated data processing and quality control are performed on the data. The automated data processing is written in Python3 (Rossum & Drake, 2009 ▸) and utilizes the package *Larch* (version 0.9.67; Newville, 2013 ▸) to load most of the data and process the data. It follows a protocol described hereafter. At first, the data provided are interpreted to extract the absorption data μ, the corresponding energy and the metadata provided. For the μ and energy extraction, file formats currently supported by *Larch*’s functions *read_ascii* and *read_spec* and text files containing μ and the energy in the first two columns are supported. For the automated extraction of the metadata, a list of supported beamlines is given in Table S2. This list is constantly updated.

In the second step, when μ and the energy are extracted, the energy position *e*0 of the edge is determined as the highest maximum of the first derivative of μ. With the determined *e*0, the absorption edge is guessed using the *Larch* function *guess_edge* in order to read out the correct energy of the edge from the Elam database (Elam *et al.*, 2002 ▸) using the *Larch* function *xray_edge*. With this the measurement energy is calibrated. In a third step, a pre- and post-edge polynomial estimation and normalization are performed using the *Larch* function *pre_edge*. This delivers the pre-processed normalized μ(*E*) and the edge step as a defined quality criteria. The edge step and a plot of the normalized spectrum are stored in the database and displayed on the front-end. In the next step, the background of the post-edge is estimated and subtracted from μ(*E*) and χ(*k*) is calculated using the *Larch* function *autobk*, which follows the *AUTOBK* algorithm from Newville *et al.* (1993 ▸). The maximum value of *k* is a quality criterion and is stored in the database. A plot of the resulting χ(*k*) is also stored in the database and displayed on the front-end. In a final step, the Fourier transformation on χ(*k*) is performed using the *Larch* function *xftf* to retrieve χ(*R*). The lower limit of the *k* range for the Fourier transformation is set by the first root position of *k*^2^ χ(*k*) of a full oscillation after *k* = 2 Å^−1^ and the upper limit as the root position at the end of a full oscillations below *k* = 13 Å^−1^. This way only meaningful oscillations are captured. A plot of χ(*k*) is stored in the database and is displayed in the front-end. The visualization is performed using *Matplotlib* (version 3.7.1; Hunter, 2007 ▸). With the estimated quality criteria and the provided plots, the user gains a fully qualified overview of the uploaded data.

Although the procedure described is well established in the community, automated evaluation is always prone for errors. Artefacts in the data and complex samples can lead to unexpected behaviour, *e.g.* for multi-elemental samples such as alloys with several overlapping edges, in particular if *L*-edges of heavier elements are involved. In the current state of the automated quality control, the parameters are optimized to metal foils and have fixed values. They do not underlie the optimization routine. Thus, the quality control is stable and always yields the same results on the same data but is not flexible. This may result in unexpected behaviour. The user itself is enabled to check the automated data evaluation by observing the plotted and listed results. Unexpected behaviour can be filed and will be analysed and help to improve the set of parameters or an implementation of an optimization routine. The fixed parameter values of the utilized *Larch* functions can be found in Table S3 if they deviate from their default values. The final safeguard is a human curator who always checks the data before they are finally entered into the database.

The automated processing of the data has been compared with a manually performed evaluation using *ATHENA* for metal foil samples measured at the P65 beamline, PETRA III, DESY. The evaluated edge steps are listed in Table 3 ▸. The comparison is discussed in detail for Mo foil measurement. A similar comparison for other metal foils data is given in Figs. S2–S4 in the supporting information.

**Figure 2:**
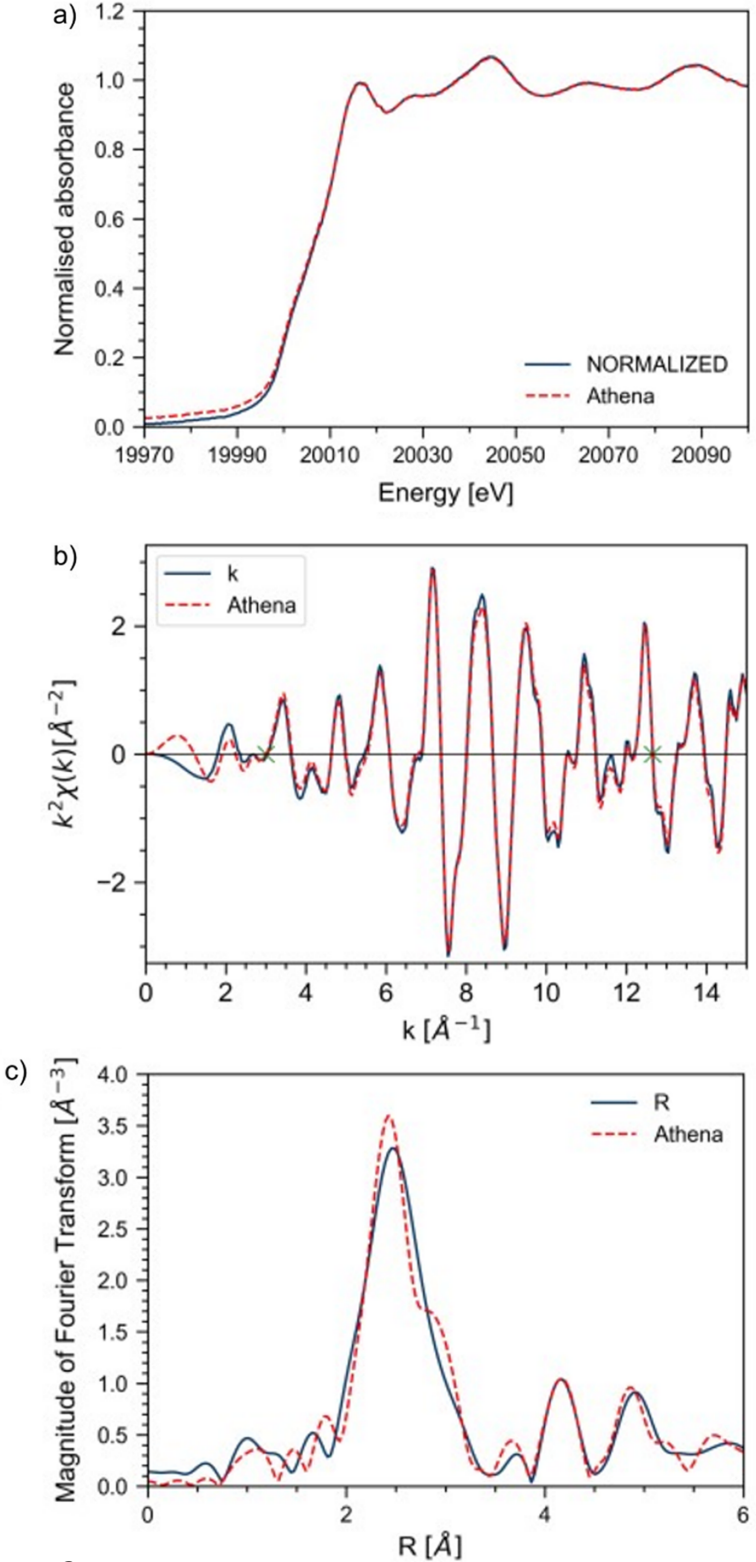
(*a*) Normalized XANES spectrum at the Mo *K*-edge for the Mo foil evaluated manually (dashed red) and automatically (blue). An overall agreement can be seen with slight deviations in the pre-edge region, resulting in a higher edge step for the automated evaluation. (*b*) Transformation in the *k* regime shows overall good agreement with slight deviations resulting from a different transformation range. (*c*) *R*-space transformation, showing a good agreement with slightly more features in the prominent peak with the manual evaluation.

For the Mo foil measurement, the automatically (blue) and manually (red dashed) evaluated plots are displayed in Fig. 2 ▸. The normalized μ(*E*) can be seen in Fig. 2 ▸(*a*). There are slight deviations in the pre-edge region, resulting in different edge steps. The edge step is 1.66 by manual evaluation and 1.63 by automated evaluation. Their deviation is less than 2%. The slight deviation directly affects the transformation in the *k* and *R* regime, resulting in slight differences in the evaluations. *k*^2^χ(*k*) is plotted in Fig. 2 ▸(*b*); higher deviations can be seen in the lower *k* range up to 2 Å^−1^, the higher part shows small deviations. The evaluated χ(*R*) is plotted in Fig. 2 ▸(*c*) and shows some deviations. Overall, more peak features are extracted with the manual evaluation, the most prominent side peak of the prominent peak is at 2.5 Å. This is not detected with the automated evaluation. Also, a slight shift of the peaks occurs with automated evaluation. Overall, the automated evaluation with its current set of parameters agrees very well with the manual evaluation and can be regarded as a good first estimation of the data quality. Nonetheless the parameter has to be always monitored and possibly adapted with more incoming data to ensure reliable and valuable data evaluation.

The stability of the procedure was tested multiple times; as there are no fit routines currently implemented, the evaluation of the loaded datasets is always reproducible.

### 2.4. Quality criteria and assessment

Evaluating the quality of the measurement data is crucial to guarantee that the derived results are accurate and reliable. In the context of reference foil samples, quality criteria considered for quality check and details about them are given in Table 4 ▸. For each criteria, a range is given in which the quality of the data is considered good.

From these quality criteria a sort of ranking can be established. The most important feature is the edge step as it directly indicates the presence of the element and the quality of the absorption data. Second comes the energy resolution which directly influences the ability to resolve fine spectral features and to interpret the data. Last comes the usable *k*-range and the amplitude reduction factor. Both are relevant for the interpretation of the data and the quality of the outcoming results. Also, the amplitude reduction factor determined from EXAFS fitting requires prior knowledge of the phase as well as the crystal structure of the sample and hence it is not possible to include this in each case. How to properly evaluate the signal-to-noise ratio is still an ongoing discussion, therefore no ranges are set. Yet there is already a first noise estimation implemented in the automated processing of the database using the *Larch* function *estimate_noise*, which estimates the noise based on the high *R* region of χ(*R*). This value is currently only accessible to the curator and is not regarded as a quality criterion.

If the criteria of the evaluated data are inside the given ranges (see Table 4 ▸), this is considered good and forwarded to the curator as such; if up to three lie outside the range still the data is forwarded to the curator and marked as such; if the data fail all parameters of quality check, then it is rejected.

In conclusion, quality assessment was a critical component of developing our reference database. By conducting a thorough evaluation of the quality of the spectra and metadata, researchers can ensure the accuracy and reliability of the data stored in the database, making it a valuable resource for the community.

Regarding access policy and human curation, at present, unrestricted access to the website is granted to all users and existing datasets can be viewed. The upload functionality is generally accessible, allowing the public to contribute datasets directly. Each dataset uploaded is licenced under ‘CC BY 4.0’ (Creative Commons, 2024 ▸) considering that this is the preferred licence under NFDI.

Contributors are required to confirm that they have read and understood the website’s disclaimer before proceeding with their upload. This confirmation is documented and stored along with the metadata in the dataset’s JSON (Pezoa *et al.*, 2016 ▸) file to ensure transparency. To maintain the high quality and integrity of the database, each dataset undergoes a human verification process in addition to automated quality control, preventing misuse and ensuring the reliability of the data provided. Finally, our human curator utilizes a sophisticated user interface on the RefXAS website to verify datasets. This interface allows the curator to examine each dataset and allows verified datasets to be displayed on the website. Additionally, the curator has the ability to alter and update metadata fields as necessary to ensure accuracy and completeness. This direct integration of the verification mask provides an efficient and user-friendly environment for the curator. Access to this verification tool is restricted to designated curators.

## 3. Technical implementation and design of the RefXAS database

**Figure 3:**
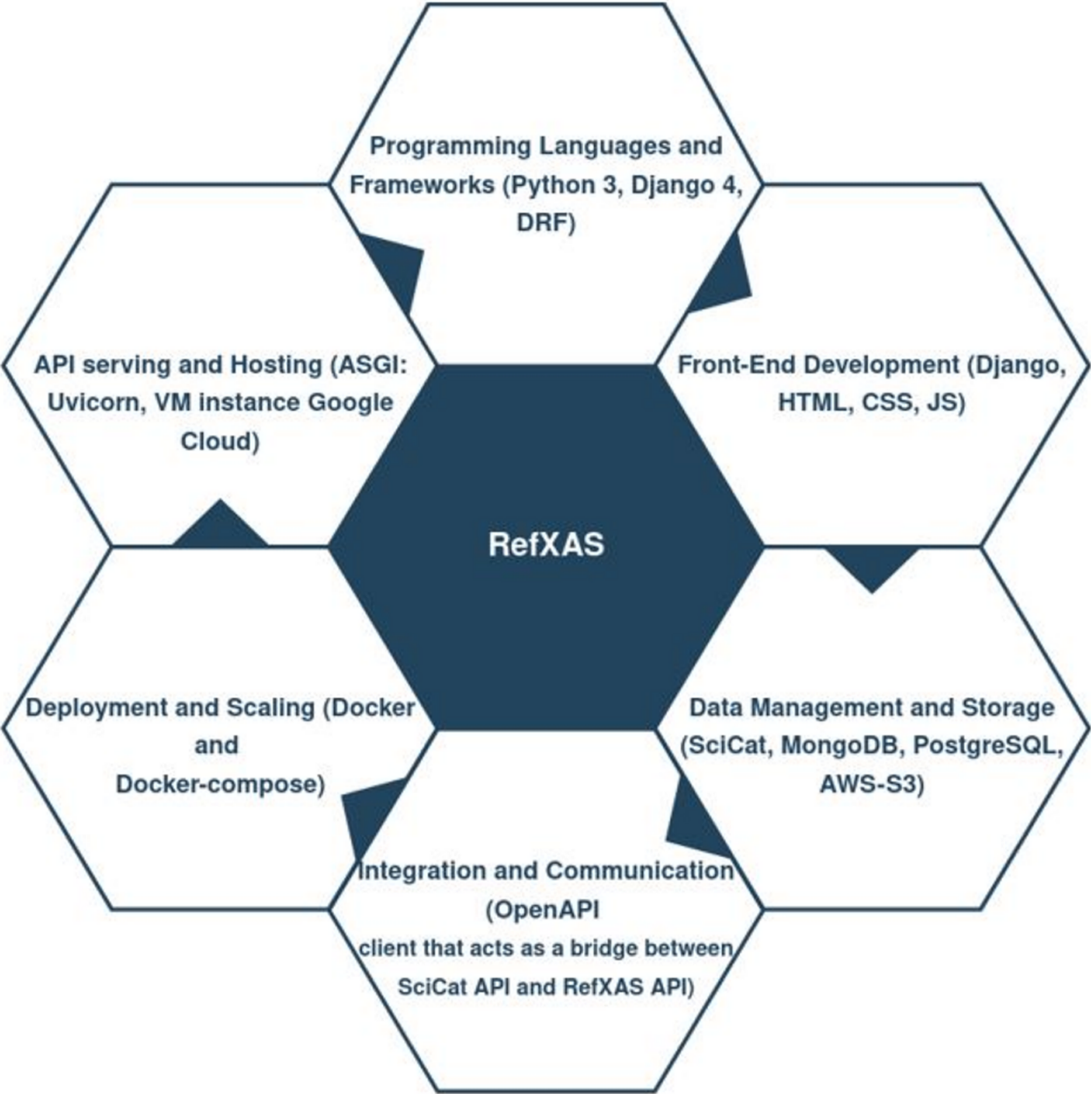
Outline of the components that represent the development of the RefXAS database. See Section S1 in the supporting information for detailed information.

The development of the RefXAS reference database required the use of various tools, programming languages, frameworks and database management systems as shown in Fig. 3 ▸.

**Figure 4:**
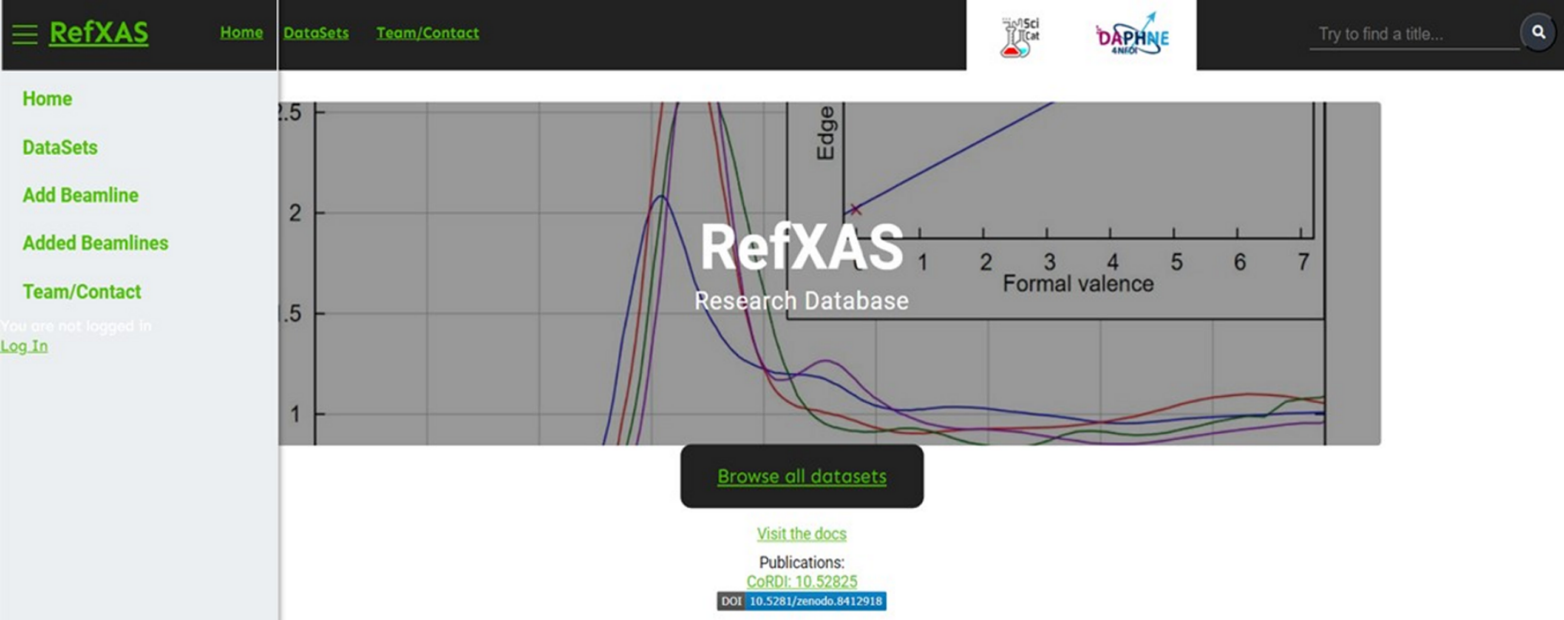
Adaptive web interface and responsive landing page of the RefXAS reference database, including the navigation bar, search bar, centrally located ‘Browse all datasets’ button and the footer.

**Figure 5:**
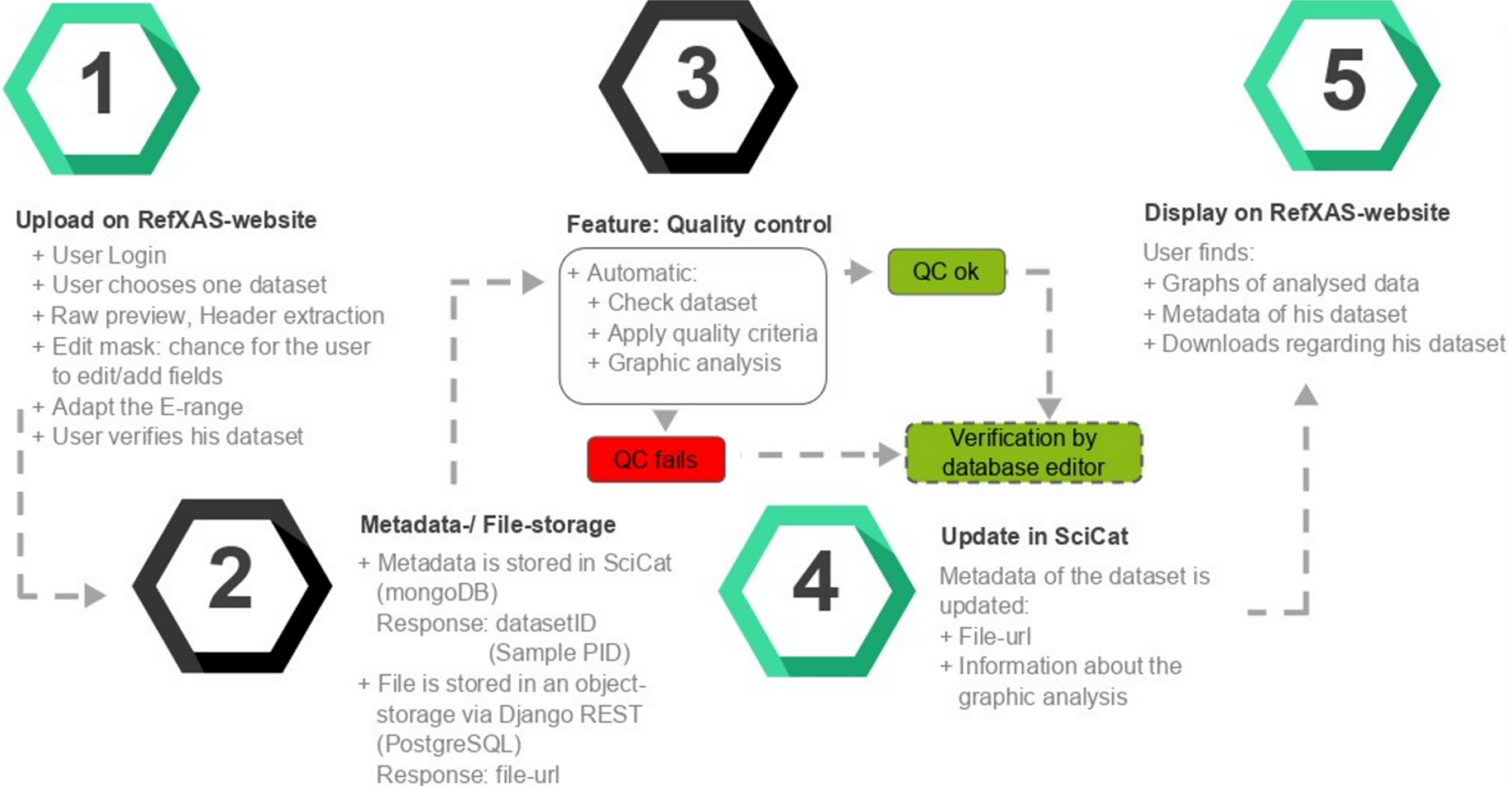
Pipeline that is executed on clicking ‘Verify and upload’. A user uploads and verifies a dataset into the pipeline at step 1. Metadata and datafile storage is step 2, and the quality control feature is illustrated in step 3. Step 4 emphasizes that the metadata are updated on dataset verification, and the dashed box indicates the human curator interaction. Step 5 shows the final result.

In the RefXAS web interface, users encounter a modern, adaptive layout on entry, featuring streamlined navigation for functions such as dataset upload and search (see Fig. 4 ▸). The interface supports comprehensive interactions like dataset queries, which can, for example, be filtered by elements or beamlines, and a direct link to the public database page (Paripsa, 2023 ▸) for enhanced user engagement. For uploading, the ‘Upload view’ simplifies dataset submission with automated scripts for data verification and metadata extraction, visualizing raw data as adjustable JPEG (Wallace, 1992 ▸) images for quality inspection. The whole pipeline is displayed in Fig. 5 ▸.

For an exhaustive description of the technical details and user interface functionalities, including data file handling and specific metadata management procedures, refer to Section S2 of the supporting information.

### 3.1. Benchmarking

To assess the performance and stability of our web server for the RefXAS database, we conducted a load test using the *Siege* tool (Fulmer, 2024 ▸). The test simulated 200 (the number of users is capped at 255) concurrent users, all with a 10 s delay between requests, sending requests over a period of 30 s to https://xafsdb.ddns.net/. This method was chosen to mimic realistic traffic patterns and determine how the server responds under high demand.

The primary goal was to quantify the capacity of the server in handling simultaneous requests, focusing on metrics such as transaction rate, response time and overall availability. These indicators are crucial for evaluating the efficiency and reliability of the server.

The test resulted in the number of maximum transactions recording at 7884 and registered a rate of the availability of the server as 100%, with all transactions being processed successfully without any failures. The server processed about 262.45 transactions per second, with an average response time of 0.28 s, an indication that it can handle quite a large number of requests within a short period. And 4.41 MB s^−1^ of data-throughput shows the large scale information transmission processing capability of the server under load. The level of concurrency was 74.60, which with the total count of successful transactions (7132) and the aggregate sum of data transferred (132.40 MB) brings out strong performance characteristics of the server. A complete list of results can be found in Table S4.

## 4. Discussion

Already in its present state, the RefXAS database appears to be well suited for practical use as a reference for users of XAS. It contains raw and processed data of well defined metal samples, along with relevant metadata about the sample and the measurements. Benchmark tests have shown that the realized front- and back-ends are able to cope with a high load of simultaneous transactions, and the user interface allows straightforward communication with the database, for the submission of both spectra and metadata, as well as for the practical use of the database for end-users. However, there are a few ideas for future optimization and development of the database, as detailed in the following sections.

### 4.1. Mandatory improvements

Our team is actively exploring and engaging in discussions around various potential enhancements aimed at further improving the database, listed below.


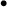
 Data management: deals with challenges so that data of all sorts of formats and datatypes could be handled automatically. We intend to extend this continuously. Additionally, we are engaged in international dialogues about adopting the NeXus data format (Könnecke *et al.*, 2015 ▸) and establishing a standard for XAS data, which will enhance our data management capabilities.


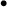
 Comparison: we intend to implement a feature allowing users to upload their datasets for server-side analysis and comparison with existing, verified datasets. This functionality will enable comparison without the necessity for dataset submission in certain scenarios, enhancing user experience and utility. Furthermore, we plan to allow users to select and compare verified and existing files from the database for analytical purposes.


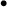
 dCache/HIFIS storage: we are in the process of transitioning from AWS S3 to a dCache storage solution provided by HIFIS at DESY. dCache offers a robust system for storing and retrieving vast amounts of data and is being further developed. It is integrated into the Helmholtz Cloud, enabling Helmholtz based user groups to store, process and publish research data. This transition marks a significant step in advancing the capabilities of the RefXAS database and aligns with our discussions with the institution to secure a long-term, stable institutional domain. This will further professionalize and stabilize the accessibility of our platform.


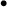
 Data retrieval/access API: the current API for data retrieval, though now used for the internal part of the web server, already has a technical foundation for use in a larger, outspread context. The architecture and important functionality components – capabilities for handling HTTP requests and structuring responses in JSON format – already exist. That is, the API already facilitates structured access to metadata, allowing users to query and retrieve specific datasets based on their criteria. Therefore, as we continue to improve our reference database, there certainly is potential for integration. To effectively employ such methodologies, the database needs to be comprehensive and robust enough to provide training data. Consequently, as our database grows and reaches a scale sufficient for this purpose, we aim to improve the development of our Data Access API. This would facilitate the extraction of data in a format suitable for machine-learning algorithms, paving the way for more sophisticated data analysis and interpretation strategies in the future.


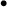
 Electronic lab-books: connection to electronic lab-books for documentation of complementary characterization, *e.g.* LabIMotion (Dolcet *et al.*, 2023 ▸). Here there are also plans to include or link complementary information on the samples such as X-ray diffraction, Raman spectroscopy *etc.* and to refer to publications detailing, for example, the properties and the preparation of the sample under investigation.

### 4.2. Future planning and long-term considerations

#### 4.2.1. Authorization API

Potential enhancement to our system could also be the implementation of an authorization API. This would facilitate the assignment of distinct roles to different user types, thus allowing us to more precisely control user access and interactions within the system. Scoping could involve the following.


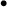
 Defining user roles: different roles could be established with varying levels of access, such as ‘administrator’, ‘contributor’ or ‘user’. Each role would have a specific scope of access and permissions. An inherent feature of Django (Django, 2023*a* ▸) is the support for an administrative (‘admin’ or ‘superuser’) role. This role, already in use, could be leveraged further to define and control distinct access levels and permissions across different user types, thus enhancing the flexibility and security of the system.


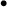
 Determining access levels: the scope of access could be determined based on factors such as the user’s role, their organization, the sensitivity of the data or other criteria.


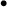
 Establishing access controls: rules or policies could be set up to control the scope of user access, such as requiring authentication or authorization, limiting access based on time or location, or using other access-control mechanisms.

#### 4.2.2. Scalability

We decided to use JSON (Pezoa *et al.*, 2016 ▸) to structure the metadata within the Django web server. This is a common practice in developing systems intended to grow and evolve over time. That is, new fields can easily be added. The implementation utilizes a dynamic table-generation approach. As each piece of metadata is stored in JSON format, it is straightforward to add additional fields into the table structure. The table is generated dynamically for each dataset, enabling the system to assist an expanding variety of data without requiring substantial code modifications. Therefore, this initial design lays a solid foundation for the anticipated growth and evolution of our reference database. For each field in the JSON object, a new cell is created in the table. This approach is scalable because adding a new field to the JSON object will simply result in an additional cell in the table, without requiring any changes to the code that generates the table. We developed the reference database with scalability in mind, that it would receive a self-accelerating effect and that the knowledge would transfer to other areas. We have been collecting high-quality data for the reference materials (*i.e.* metal foils, oxides and other compounds). The data to be uploaded have been quality-checked based on the quality criteria defined in Section 2.4.

To start, there are around 100 datasets in the ‘to be uploaded’ list, including oxides and other compounds of elements. However, the number of metal foil data which have been already quality checked is around 40. These datasets are a collaborative effort from multiple institutions and measured at different synchrotron beamlines and laboratory X-ray spectroscopy setups. In the next step, we focus on more realistic powder samples, such as pellets and capillaries, to better represent the experimental conditions commonly encountered in practical applications. This stage will involve the inclusion of reference compounds which are frequently studied in the field. By expanding the database to cover these more complex and realistic sample types, we will aim to enhance its applicability and utility for researchers.

After successfully developing the interface and uploading metal foil references in the initial phase, we plan to focus on Fe *K*-edge and Cu *K*-edge data, as these elements play an important role in catalytic reactions and are extensively reported in the literature. By incorporating a wide range of data for Fe and Cu, the reference database would serve as a valuable resource for researchers in the field of catalysis and XAS, enabling the comparison and interpretation of experimental results with greater accuracy and confidence.

#### 4.2.3. Long-term deployment possibility

Currently, our reference database is hosted on a Google virtual machine utilizing *Docker Compose*, complemented by AWS-S3 and a registered domain. This setup has proven to be effective during our initial development and testing phases. However, for the purpose of long-term deployment, scalability and independence from a single provider, we are actively exploring several strategies to enhance our infrastructure. Hence, we are in the process of identifying the most suitable alternatives. We are aware of the challenges and difficulties in moving to a new deployment architecture. Nevertheless, we are committed to make this transition strategically, with long-term sustainability and scalability in mind. Although this is a complex process requiring extensive considerations, we realized that this is an essential step towards ensuring the future growth, success and sustainability of our reference database.

#### 4.2.4. User effort

We strongly believe that the reference database belongs to the user community. As such, we would highly value and invite all forms of feedback, suggestions and contributions. Our aim is to encourage a dynamic, collaborative environment, where users actively shape and enhance the system in line with their evolving needs and interests. On this matter, we invite users to fill in the contact form on our website to provide feedback.

## 5. Conclusions

The development of the RefXAS database represents a significant milestone in the field of XAS. We have carefully established a robust and comprehensive system for managing, storing and analysing XAS data. Our approach prioritizes the inclusion of high-quality real spectra and metadata, initially focusing on metal foils due to their stability and reproducibility. Plans are underway to extend the database to accommodate more diverse sample types, thereby enhancing its applicability across a broad spectrum of research areas.

Overall, the RefXAS reference database is a powerful and flexible tool that provides a comprehensive solution for providing quality-checked XAS spectra with indexed metadata along with pre-processing tools for visualization and comparison of XAS data across facilities and laboratory setups, respectively, making it an attractive option for researchers and practitioners in the field. In a second stage, the inclusion of X-ray emission spectroscopic data is envisaged.

The technical infrastructure ensures the scalability and resilience of the database. We have successfully integrated functionalities such as automated quality control, dynamic metadata field handling and a user-friendly interface, making the database accessible to researchers with varied technical backgrounds. Additionally, we are exploring potential enhancements such as a Data Access API for machine-learning applications and an Authorization API for clarified user role management. Looking ahead, we acknowledge the need for strategic planning and implementation to ensure the long-term sustainability and evolution of the RefXAS database. As we continue to advance this project, we remain committed to contributing significantly to the standardization and enhancement of XAS data management and analysis, thereby serving as a valuable resource for the scientific community.

## 6. Related literature

The following references, not cited in the main body of the paper, have been cited in the supporting information: Amazon (2023 ▸); Django (2023*b* ▸); Google (2023 ▸); Merkel (2014 ▸); MongoDB (2023 ▸); OpenAPI (2023 ▸); Pithan (2022 ▸, 2023 ▸); PostgreSQL (2023 ▸); Uvicorn (2023 ▸); YAML (2023 ▸).

## Supplementary Material

Supporting figures and tables. DOI: 10.1107/S1600577524006751/up5002sup1.pdf

## Figures and Tables

**Table 1:** Overview of all defined (meta)data fields All metadata fields are stored in the format of a JSON (Pezoa *et al.*, 2016 ▸) dictionary (see section S1 of the supporting information for more information).

(Meta)data field	Description/example
**Sample**	
Sample ID	Provides a unique identifier for each sample and process
Collection code	Chemical symbol of element with edge (*K*, *L*, *Metc*.) and scan number
CAS No. (if available)	Standardized identifier for chemical compounds
Physical state	*E.g.* crystalline, powder, thin film, liquid, gas
Structural parameters for crystalline samples	Can affect the diffraction pattern, for now only crystalline or single-phase samples can be used, in future we aim for more complex systems
Crystal orientation	*E.g.* Miller indices
X-ray or neutron diffractogram (if available)	Provides information about the crystal structure
Temperature, pressure and environment	Can impact the XAS spectrum
Remarks about sample preparation	*E.g.* foil, pellet, capillary, powder on tape
Sample environment	*E.g.* cell/microreactor/batch, gases, solvents, potential

**Spectra**	
Raw data file	Provides the original data acquired by the instrument
Raw absorbance spectrum	Processed XAS spectrum
Normalized absorbance spectrum	Normalized XAS spectrum/XANES
χ(*k*)	Represents the extended X-ray absorption fine structure (EXAFS) oscillations in *k*-space (wavevector space)
χ(*R*)	Represents the EXAFS oscillations in *R*-space (radial distance space), obtained by performing the Fourier transform of the EXAFS oscillations in *k*-space without employing corrections for phase shifts
Reference spectrum†	If available, used as a standard to check the energy scale calibration and for comparing beamline parameters

**Instrument**	
Facility	Whether synchrotron or laboratory instrument
Beamline	*E.g.* P65, CAT-ACT *etc.*, preferentially a DOI
Acquisition mode	Refers to the way in which data are collected from the sample (*e.g.* ‘continuous scan’, ‘step-scan’ or ‘energy-dispersive’ measurement mode), absorption, fluorescence or total electron yield mode
Crystals	*E.g.* Fe_2_O_3_ (hematite), DCM, CCM, polychromator, specify materials and orientation [*e.g.* Si(111), Ge(220), *etc.*]
Mirrors	Optical devices that are used to focus, collimate or deflect X-ray beams; specify mirror material, incident angle and form of the mirror (plane, bent)
Detectors	Devices used to measure and record the intensity of X-rays that have interact with a sample during an experiment, *e.g.* gas-filled ionization chambers, solid-state detectors
Element	*E.g.* Fe
Absorption edge	*E.g.K*-edge
Maximum *k* range (EXAFS)	Refers to the upper limit of the wavevector (*k*) values considered during the analysis of the EXAFS oscillations
Resolution	Energy resolution, which is the ability of a spectrometer to distinguish between two closely spaced X-ray photon energies

**Bibliography**	
DOI	Identifier
Title	*e.g.* <sample-name> + <time-stamp>
Author	<Full name>
Literature reference	Provides more information about the data

† Reference spectrum, for example, of a metal foil or available standard compound of the element measured simultaneously along with the sample.

**Table 2:** Structural parameters obtained from EXAFS fitting for the Mo foil measurements *N* = coordination number, *R* = bond distance, σ^2^ = Debye–Waller factor, ‘*R* factor’ = goodness of fit parameter. The error bars are given in parenthesis.

Mo foils (distinct) measured at XAS beamlines from different synchrotron facilities										
Beamline	*N*	*R* (Å)	σ^2^ × 10^3^ (Å^2^)	*N*	*R* (Å)	σ^2^ × 10^3^ (Å^2^)	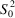	Δ*E*	*R* factor	*k* range for FT
1	8	2.72 (0.01)	3.6 (0.4)	6	3.14 (0.01)	3.6 (0.5)	0.97 (0.07)	4.9 (0.8)	0.0006	2.2–15.2
2	8	2.72 (0.01)	3.7 (0.3)	6	3.14 (0.01)	3.7 (0.4)	0.98 (0.06)	5.2 (0.7)	0.0004	2.2–15.2
3	8	2.73 (0.01)	4.0 (0.2)	6	3.15 (0.01)	3.9 (0.3)	1.03 (0.05)	5.5 (0.5)	0.0004	2.2–18.0
4	8	2.72 (0.01)	3.5 (0.3)	6	3.14 (0.01)	3.6 (0.4)	0.94 (0.05)	5.1 (0.7)	0.0004	2.2–15.2
5	8	2.72 (0.01)	3.8 (0.2)	6	3.14 (0.01)	3.9 (0.3)	0.97 (0.04)	4.8 (0.5)	0.0003	2.2–18.0

**Table 3:** Evaluated edge steps from a manual and automated evaluation of single-element foil measurements

Foil	Manual evaluation	Automated evaluation	Deviation (%)
Au	1.80	1.80	0
Fe	2.33	2.33	0
Mo	1.66	1.63	1.8
Ni	1.33	1.33	0
Pt	0.85	0.84	1.2
Rh	1.90	1.98	4
Ru	1.65	1.65	0
Zn	1.88	1.88	0

**Table 4:** Brief overview of the quality criteria for foil samples on synchrotron measurements

Quality criteria	Range	Description
Edge step (transmission mode)	0.5–2.0	Refers to the steepness of the onset of the absorption edge and is an indicator of the quality of the sample and the measurement. A high edge step, characterized by a steep onset of the absorption edge, is preferred as it reduces background noise in the EXAFS data and improves the accuracy of the measurement results.
Maximum usable *k*-range	15–20 Å^−1^	Refers to the range of wavevector (*k*) values used to examine the EXAFS oscillations. The choice of *k*-range directly impacts data quality, structural resolution and the accuracy of derived results.
Energy step or point density as calculated from spectral points	0.5–2 eV	Refers to sampling frequency in the energy domain in the XANES region. Energy step or point density should not be confused with energy resolution mentioned in Table 1 ▸ which is the ability of a spectrometer to distinguish between two closely spaced X-ray photon energies.
Amplitude reduction factor ( 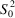 )†	0.7–1.0	Correction factor that accounts for the reduction in the EXAFS oscillation amplitude. Essential for accurately determining the coordination numbers from the EXAFS data.
Signal-to-noise ratio	0.1% of edge jump	Quantifies the proportion of meaningful signal to the background noise present in the data.

†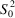 is determined after fitting the model to the EXAFS data and here this parameter is mentioned specifically for the case of metal foils where the structure is known in most cases.

## Data Availability

The authors confirm that the data supporting the findings of this study are available within the article and its supplementary materials.

## References

[bb1] Amazon (2023). *Amazon S3 storage. Amazon Web Services*, https://aws.amazon.com/de/s3/.

[bb2] Asakura, K., Abe, H. & Kimura, M. (2018). *J. Synchrotron Rad.* **25**, 967–971. 10.1107/S1600577518006963 PMC6038598 29979157

[bb3] Ascone, I., Asakura, K., George, G. N. & Wakatsuki, S. (2012). *J. Synchrotron Rad.* **19**, 849–850. 10.1107/S0909049512043506 23093741

[bb4] Barty, A., Gutt, C., Lohstroh, W., Murphy, B., Schneidewind, A., Grunwaldt, J.-D., Schreiber, F., Busch, S., Unruh, T., Bussmann, M., Fangohr, H., Görzig, H., Houben, A., Kluge, T., Manke, I., Lützenkirchen-Hecht, D., Schneider, T. R., Weber, F., Bruno, G. & Turchinovich, D. (2023). *DAPHNE4NFDI – Consortium Proposal*, https://doi.org/10.5281/zenodo.8040606.

[bb5] Bertagnolli, H. (1989). *Ber. Bunsenges. Phys. Chem.* **93**, 229.

[bb6] Boyanov, B. & Segre, C. (1995). *Farrel Lytle Database*, https://ixs.iit.edu/database/data/Farrel_Lytle_data/.

[bb7] Calvin, S. (2013). *XAFS for Everyone*, 1st ed. CRC Press.

[bb8] Chantler, C. T., Bunker, B. A., Abe, H., Kimura, M., Newville, M. & Welter, E. (2018). *J. Synchrotron Rad.* **25**, 935–943. 10.1107/S1600577518003752 PMC6038608 29979153

[bb9] Chen, Y., Chen, C., Zheng, C., Dwaraknath, S., Horton, M. K., Cabana, J., Rehr, J., Vinson, J., Dozier, A., Kas, J. J., Persson, K. A. & Ong, S. P. (2021). *Sci. Data*, **8**, 153. 10.1038/s41597-021-00936-5 PMC8196187 34117266

[bb10] Cibin, G., Gianolio, D., Parry, S. A., Schoonjans, T., Moore, O., Draper, R., Miller, L. A., Thoma, A., Doswell, C. L. & Graham, A. (2020). *Radiat. Phys. Chem.* **175**, 108479.

[bb11] Creative Commons BY-NC-SA (2024). *Attribution-NonCommercial-ShareAlike 4.0 International*, https://creativecommons.org/licenses/by-nc-sa/4.0/.

[bb12] Django (2023*a*). *Django Software Foundation*, https://www.djangoproject.com/foundation/.

[bb13] Django (2023*b*). *Django REST Framework. Encode OSS*, https://www.django-rest-framework.org/.

[bb14] Dolcet, P., Schulte, M. L., Maurer, F., Jung, N., Chacko, R., Deutschmann, O. & Grunwaldt, J.-D. (2023). *1st Conference on Research Data Infrastructure (CoRDI) – Connecting Communities*, 12–14 September 2023, Karlsruhe, Germany, edited by Y. Sure-Vetter & C. Goble.

[bb15] Doronkin, D. E., Casapu, M. & Grunwaldt, J.-D. (2020). *Synchrotron Radiat. News*, **33**(5), 11–17.

[bb17] Elam, W. T., Ravel, B. D. & Sieber, J. R. (2002). *Radiat. Phys. Chem.* **63**, 121–128.

[bb18] Frahm, R. (1989). *Rev. Sci. Instrum.* **60**, 2515–2518.

[bb19] Frenkel, A. I., Khalid, S., Hanson, J. C. & Nachtegaal, M. (2013). *In-situ Characterization of Heterogeneous Catalysts*, edited by J. A. Rodriguez, J. C. Hanson & P. J. Chupas, pp. 23–47. New York: Wiley.

[bb20] Fulmer, J. (2024). *Siege*, https://www.joedog.org/siege-home/.

[bb21] Gaur, A., Paripsa, S., Förste, F., Doronkin, D., Malzer, W., Schlesiger, C., Kanngießer, B., Lützenkirchen-Hecht, D., Welter, E. & Grunwaldt, J.-D. (2023). *1st Conference on Research Data Infrastructure (CoRDI) – Connecting Communities*, 12–14 September 2023, Karlsruhe, Germany, edited by Y. Sure-Vetter & C. Goble.

[bb22] Gaur, A. & Shrivastava, B. D. (2015). *Ref. J. Chem.* **5**, 361–398.

[bb23] Gaur, A., Shrivastava, B. D. & Nigam, H. (2013). *Proc. Indian Natl. Sci. Acad.* **79**, 921–966.

[bb24] George, G. N. & Pickering, I. J. (2013). *Encyclopedia of Biophysics*, edited by G. C. K. Roberts, pp. 2762–2767. Berlin, Heidelberg: Springer.

[bb25] Google (2023). *Google Cloud – Virtual Machine Instances*, https://cloud.google.com/products/compute.

[bb26] Hunter, J. D. (2007). *Comput. Sci. Eng.* **9**, 90–95.

[bb29] Isaure, M.-P., Laboudigue, A., Manceau, A., Sarret, G., Tiffreau, C., Trocellier, P., Lamble, G., Hazemann, J.-L. & Chateigner, D. (2002). *Geochim. Cosmochim. Acta*, **66**, 1549–1567.

[bb30] Ishii, M., Tanabe, K., Matsuda, A., Ofuchi, H., Matsumoto, T., Yaji, T., Inada, Y., Nitani, H., Kimura, M. & Asakura, K. (2023). *Sci. Technol. Adv. Mater.* **3**, 2197518.

[bb31] Jain, A., Ong, S. P., Hautier, G., Chen, W., Richards, W. D., Dacek, S., Cholia, S., Gunter, D., Skinner, D., Ceder, G. & Persson, K. A. (2013). *APL Mater.* **1**, 011002.

[bb32] Kelly, S. D., Bare, S. R., Greenlay, N., Azevedo, G., Balasubramanian, M., Barton, D., Chattopadhyay, S., Fakra, S., Johannessen, B., Newville, M., Pena, J., Pokrovski, G. S., Proux, O., Priolkar, K., Ravel, B. & Webb, S. M. (2009). *J. Phys. Conf. Ser.* **190**, 012032.

[bb33] Kieffer, I. & Testemale, D. (2016). *SSHADE: the Solid Spectroscopy database infrastructure*, https://www.sshade.eu/doi/10.26302/SSHADE/FAME.

[bb34] Könnecke, M., Akeroyd, F. A., Bernstein, H. J., Brewster, A. S., Campbell, S. I., Clausen, B., Cottrell, S., Hoffmann, J. U., Jemian, P. R., Männicke, D., Osborn, R., Peterson, P. F., Richter, T., Suzuki, J., Watts, B., Wintersberger, E. & Wuttke, J. (2015). *J. Appl. Cryst.* **48**, 301–305. 10.1107/S1600576714027575 PMC4453170 26089752

[bb35] Lamberti, C. & van Bokhoven, J. A. (2016). *X-ray Absorption and X-ray Emission Spectroscopy: Theory and Applications*, edited by J. A. Van Bokhoven & Carlo Lamberti, pp. 351–383. New York: John Wiley & Sons.

[bb36] Lehnert, K., Klump, J., Ramdeen, S., Wyborn, L. & Haak, L. (2021). *IGSN 2040 Summary Report: Defining the Future of the IGSN as a Global Persistent Identifier for Material Samples*, https://doi.org/10.5281/zenodo.5118289.

[bb37] Mathew, K., Zheng, C., Winston, D., Chen, C., Dozier, A., Rehr, J. J., Ong, S. P. & Persson, K. A. (2018). *Sci. Data*, **5**, 180151. 10.1038/sdata.2018.151 PMC6067047 30063226

[bb38] Merkel, D. (2014). *Linux J.* **2014**(239), 2.

[bb39] Meyer, R. J., Bare, S. R., Canning, G. A., Chen, J. G., Chu, P. M., Hock, A. S., Hoffman, A. S., Karim, A. M., Kelly, S. D., Lei, Y., Stavitski, E. & Wrasman, C. J. (2024). *J. Catal.* **432**, 115369.

[bb40] MongoDB (2023). *MongoDB*, https://www.mongodb.com/de-de.

[bb41] Müller, O., Lützenkirchen-Hecht, D. & Frahm, R. (2015). *Rev. Sci. Instrum.* **86**, 035105. 10.1063/1.4913900 25832273

[bb42] Newville, M. (2013). *J. Phys. Conf. Ser.* **430**, 012007.

[bb43] Newville, M., Līviņš, P., Yacoby, Y., Rehr, J. J. & Stern, E. A. (1993). *Phys. Rev. B*, **47**, 14126–14131. 10.1103/physrevb.47.14126 10005753

[bb44] NeXus (2024). *NeXus*, https://www.nexusformat.org/.

[bb45] Ofuchi, H., Matsumoto, T. & Honma, T. (2024). *Radiat. Phys. Chem.* **218**, 111581.

[bb62] OpenAPI (2023). *OpenAPI*, https://www.openapis.org/.

[bb46] Paripsa, S. (2023). *RefXAS – Reference database for XAS*, https://san-wierpa.github.io/xafsdb_webserver/.

[bb47] Pezoa, F., Reutter, J. L., Suarez, F., Ugarte, M. & Vrgoč, D. (2016). *Foundations of JSON Schema, Proceedings of the 25th International Conference on World Wide Web (WWW’16)*, 11–15 April 2016, Montréal, Québec, Canada, pp. 263–273. International World Wide Web Conferences Steering Committee.

[bb48] Pithan, L., Jordt, P., Pylypenko, A., Richter, T., Schreiber, F. & Murphy, B. (2022). *SciCat: Implementing a data catalogue for individual research groups*, https://dx.doi.org/10.13140/RG.2.2.26963.66080.

[bb49] Pithan, L., Novelli, M., McReynolds, D., Shemilt, L., Minotti, C., Pylypenko, A., Gerlach, A., Hinderhofer, A., Egli, S., Richter, T. & Schreiber, F. (2023). *SciCat: A meta data catalog and research data management system*, https://dx.doi.org/10.13140/RG.2.2.19320.72967.

[bb50] PostgreSQL (2023). *PostgreSQL: The World’s Most Advanced Open Source Relational Database*, https://www.postgresql.org/.

[bb51] Q2XAFS (2023). *Q2XAFS 2023 | International Workshop on Improving Data Quality and Quantity in XAFS Spectroscopy*, https://www.ansto.gov.au/whats-on/q2xafs-2023-international-workshop-on-improving-data-quality-and-quantity-xafs.

[bb52] Ravel, B., Hester, J. R., Solé, V. A. & Newville, M. (2012). *J. Synchrotron Rad.* **19**, 869–874. 10.1107/S0909049512036886 23093744

[bb53] Ravel, B. & Newville, M. (2005). *J. Synchrotron Rad.* **12**, 537–541. 10.1107/S0909049505012719 15968136

[bb54] Ravel, B. & Newville, M. (2016). *J. Phys. Conf. Ser.* **712**, 012148. 10.1088/1742-6596/712/1/012148 PMC4971576 27499797

[bb55] Rehr, J. J., Kas, J. J., Vila, F. D., Prange, M. P. & Jorissen, K. (2010). *Phys. Chem. Chem. Phys.* **12**, 5503–5513. 10.1039/b926434e 20445945

[bb56] Ressler, T., Brock, S. L., Wong, J. & Suib, S. L. (1999). *J. Phys. Chem. B*, **103**, 6407–6420.

[bb57] Rossberg, A. S. A. C., Schmeisser, N., Rothe, J., Kaden, P., Schild, D., Wiss, T. & Daehn, R. (2014). *AcReDaS Actinide reference database for Spectroscopy (formerly AcXAS)*, https://www.hzdr.de/acredas.

[bb58] Rossum, G. V. & Drake, F. L. (2009). *Python 3 Reference Manual.* CreateSpace.

[bb59] Sayers, D. E. (2000*a*). *Report of the International XAFS Society Standards and Criteria Committee*, pp. 1–15, https://docs.xrayabsorption.org/StandardsCriteria_Reports/StandardsCriteria_2000.pdf.

[bb61] Stötzel, J., Lützenkirchen-Hecht, D. & Frahm, R. (2010). *Rev. Sci. Instrum.* **81**, 073109. 10.1063/1.3458015 20687707

[bb63] Timoshenko, J. & Roldan Cuenya, B. (2021). *Chem. Rev.* **121**, 882–961. 10.1021/acs.chemrev.0c00396 PMC7844833 32986414

[bb64] Uvicorn (2023). *Uvicorn*, https://www.uvicorn.org/.

[bb65] Wallace, G. K. (1992). *IEEE Trans. Consum. Electron.* **38**, xviii–xxxiv.

[bb66] Wasserman, S. R., Allen, P. G., Shuh, D. K., Bucher, J. J. & Edelstein, N. M. (1999). *J. Synchrotron Rad.* **6**, 284–286. 10.1107/S0909049599000965 15263280

[bb67] Wu, Y., Tang, X., Zhang, F., Li, L., Zhai, W., Huang, B., Hu, T., Lützenkirchen-Hecht, D., Yuan, K. & Chen, Y. (2022). *Mater. Chem. Front.* **6**, 1209–1217.

[bb70] YAML (2023). *The YAML Project*, https://yaml.org/.

