# Supplementary material for: RefXAS: an open access database of X-ray absorption spectra
Source: J Synchrotron Radiat. 2024 Aug 27;31(Pt 5):1105–17. doi: 10.1107/S1600577524006751 (PMC11371060; doi:10.1107/S1600577524006751)
Supplement: Supplementary file 1 [file s-31-01105-sup1.pdf]

JOURNAL OF SYNCHROTRON RADIATION

Volume 31 (2024)

Supporting information for article:

RefXAS: An Open Access database of X-ray absorption spectra

Sebastian Paripsa, Abhijeet Gaur, Frank Förste, Dmitry E. Doronkin, Wolfgang Malzer, Christopher Schlesiger, Birgit Kanngießer, Edmund Welter, Jan-Dierk Grunwaldt and Dirk Lützenkirchen-Hecht

Figure S1 Details of different steps involved in catalyst sample's life cycle, i.e., synthesis, testing and characterisation showing presence of catalyst in different sample forms at these steps.

(Arrows indicate exchange of sample information at different steps)

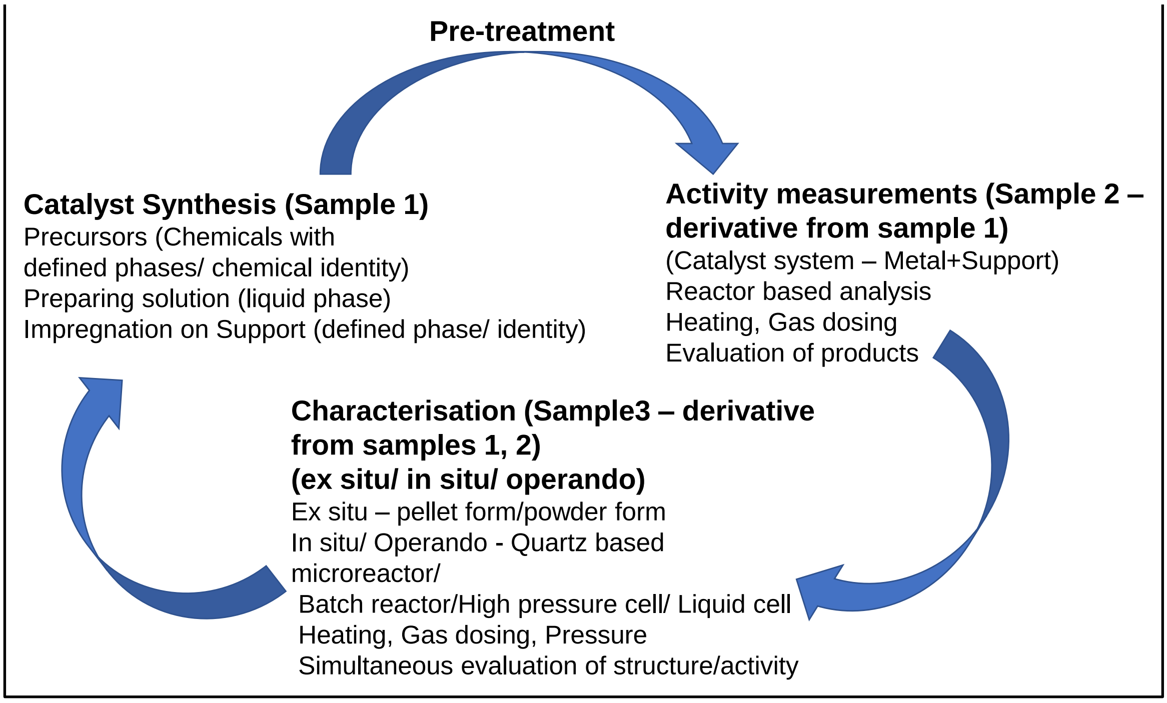

Table S1 Overview of the defined metadata fields for a beamline.

Beamline metadata field	Description / Example
**Facility**	
Synchrotron name	identifies the specific synchrotron facility where the research is conducted, with corresponding PID (*The Research Organization Registry (ROR)* 2023)
Beamline	refers to the specific beamline used at the synchrotron, DOI of description if available
**Storage ring**	
Energy	indicates the energy level of the electron beam in the storage ring. Unit: GeV
Beam emittance	describes the quality of the electron beam. unit: nmrad
Filling mode	refers to the pattern in which electrons are distributed in the storage ring
**Mirrors**	
Use mirror	Yes, No
Position	before / after monochromator, both
Reflecting surface material	specifies the material composition of the mirror's reflecting surface.
Angle of incidence	refers to the angle at which the synchrotron light strikes the mirror. Unit: mrad
**Filters / windows**	
Filter	specifies the type and characteristics of the filter used in the beamline.

windows	describes the material and specifications of the windows in the beam-line.
**Detectors**	
If IC used gases and pressure (IC: Ionization chamber)	separate fields for I0 and I1 (I0: Ionization chamber before the sample, I1: Ionization chamber after the sample)
If fluorescence detection	type of detection
**Scan parameter**	
Detection mode	identifies the method used for detecting the synchrotron radiation. e.g. Fluorescence, Transmission, electron yield
Scan mode	refers to the technique employed for scanning the sample with the beam. E.g. Continuous, Steps
Monochromatic flux on sample	indicates the intensity of the monochromatic beam as it interacts with the sample.
Beamsize on sample	specifies the size of the beam when it hits the sample.
Higher harmonic content of beam	actual, measured
**Source-type**	
Type	undulator (tapered, scanned, …), Wiggler, Bending magnet
Critical energy	indicates the energy at which the intensity of the synchrotron radiation is at its peak. Unit: eV
Maximum k value	refers to the highest wavevector value achievable. Integer (in $Å^{-1}$)
**Monochromator**	
Type	design and functional type of the monochromator. e.g. DCM (Double crystal monochromator)
Crystals	e.g. Fe2O3 (hematite)DCM, CCM, Polychromator, specify materials and orientation, e.g. Si (111), Ge (220), etc.
Lattice spacing	indicates the spacing between atoms in the monochromator crystals. Used to calculate the energy axis
Temperature of crystals	refers to the operational temperature of the monochromator crystals. Unit: K
Distances: Source to DCM	e.g. (in Meters)
Distances: DCM to sample	e.g. (in Meters)
Encoder on theta axis	either No, or Yes -> Resolution (angle units)
Position of slits	distance from source or sample
Opening of slits	describes the aperture size of the slits in the monochromator setup
Energy resolution	indicates the precision with which the monochromator can select specific energy wavelengths of the X-ray beam
Detuning	refers to the deliberate misalignment of the monochromator crystals to reduce the intensity of higher-order harmonics. Unit: percentage of maximum intensity
**Beam damage (**If applicable**)**	
No	NaN
Yes	details

Table S2 The table gives an overview of the up-to-date supported synchrotron beamlines for automated metadata extraction.

**Supported beamlines for automated metadata extraction**
**CATACT KIT**
**PETRA III Extension Beamline P65**
**ELETTRA XAFS**
**SLRI**
**ESRF BM 23**
**SOLEIL ROCK**
**SOLEIL SAMBA**
**SLS**
**DELTA**
**SOLARIS**

Table S3 Listed are the parameters and their assigned values if they deviate from their default values of the Larch functions utilized in the automated data processing.

**Larch function**	**Parameters**
**pre_edge**	*pre1 = -150*<br>*pre2 = -30*<br>*norm1 = 50*<br>*norm2 = 700*<br>*make_flat = True*<br>*nvict = 3*
**autobk**	clamp_lo = 10<br>clamp_hi = 1<br>dk = 1<br>kweight = 2
**xftf**	kmin = first root of oscillation after k = 2 $Å^{-1}$<br>kmax = root of full oscillation before k = 13 $Å^{-1}$ kweight = 2<br>window = 'hanning'<br>rmax_out = 12,

Figure S2 EXAFS fittings for the Mo foils **1–5** in R-space. The experimental curve (black solid line) along with the theoretical fit (red dashed line) are shown in the figures.

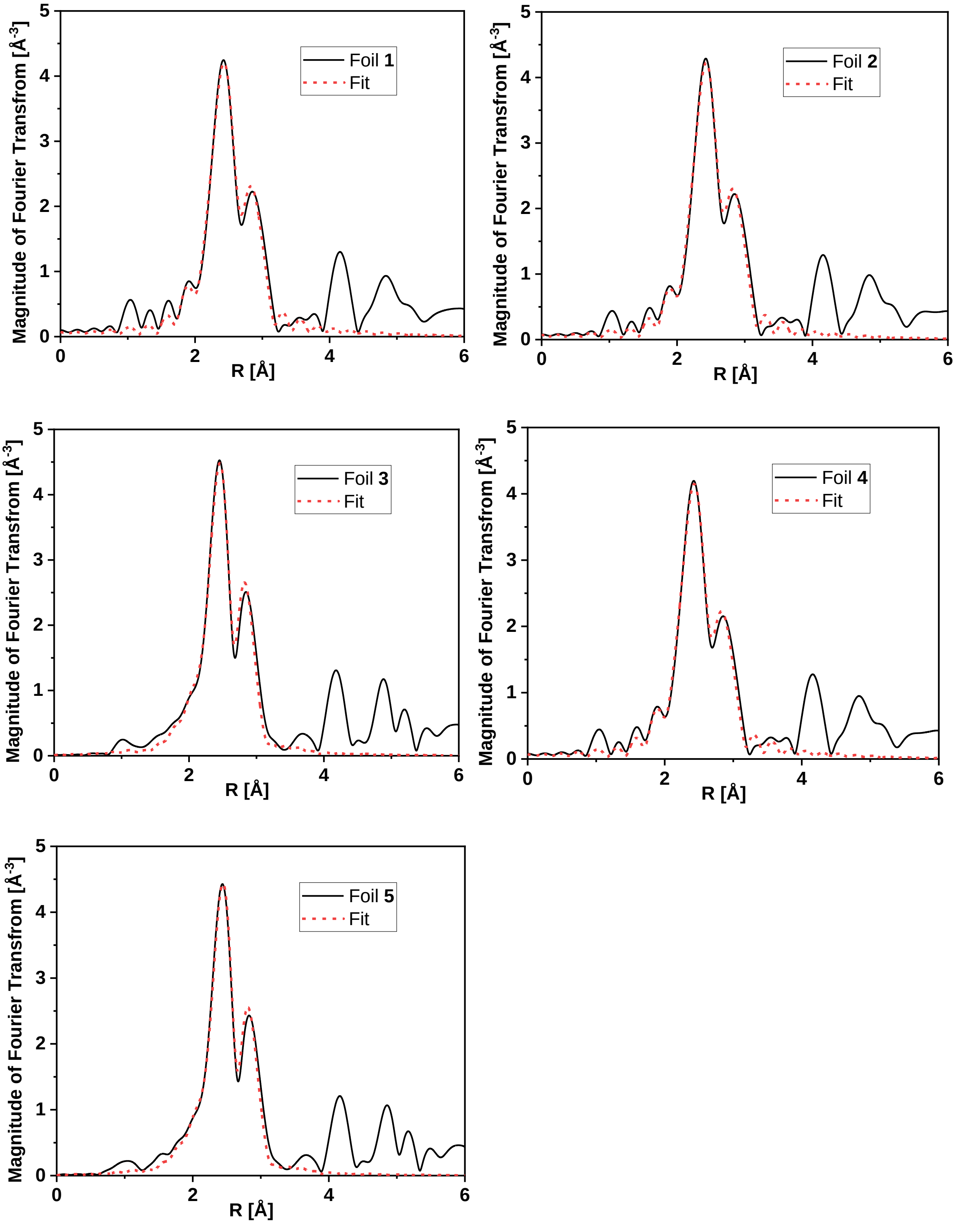

Figure S3 Comparison of normalized EXAFS absorption spectra for the metal foils. The red dashed line represents the manually evaluated spectra using the software *Athena* the blue line the automatically evaluated spectra.

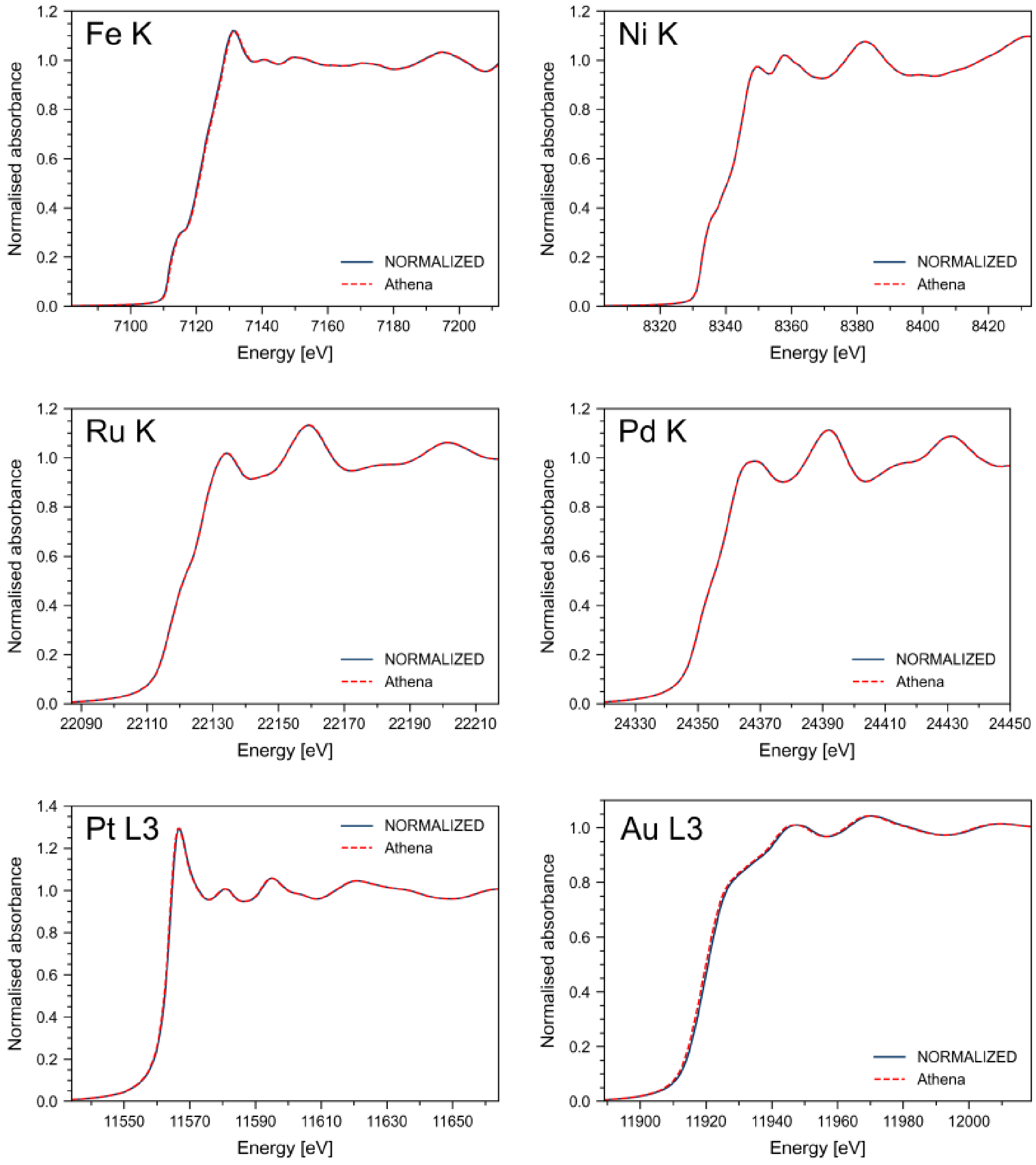

Figure S4 Comparison of k^2-weighted $\chi(k)$ spectra the metal foils in k-space. The red dashed line represents the manually evaluated spectra using the software *Athena* and the blue line the automatically evaluated spectra.

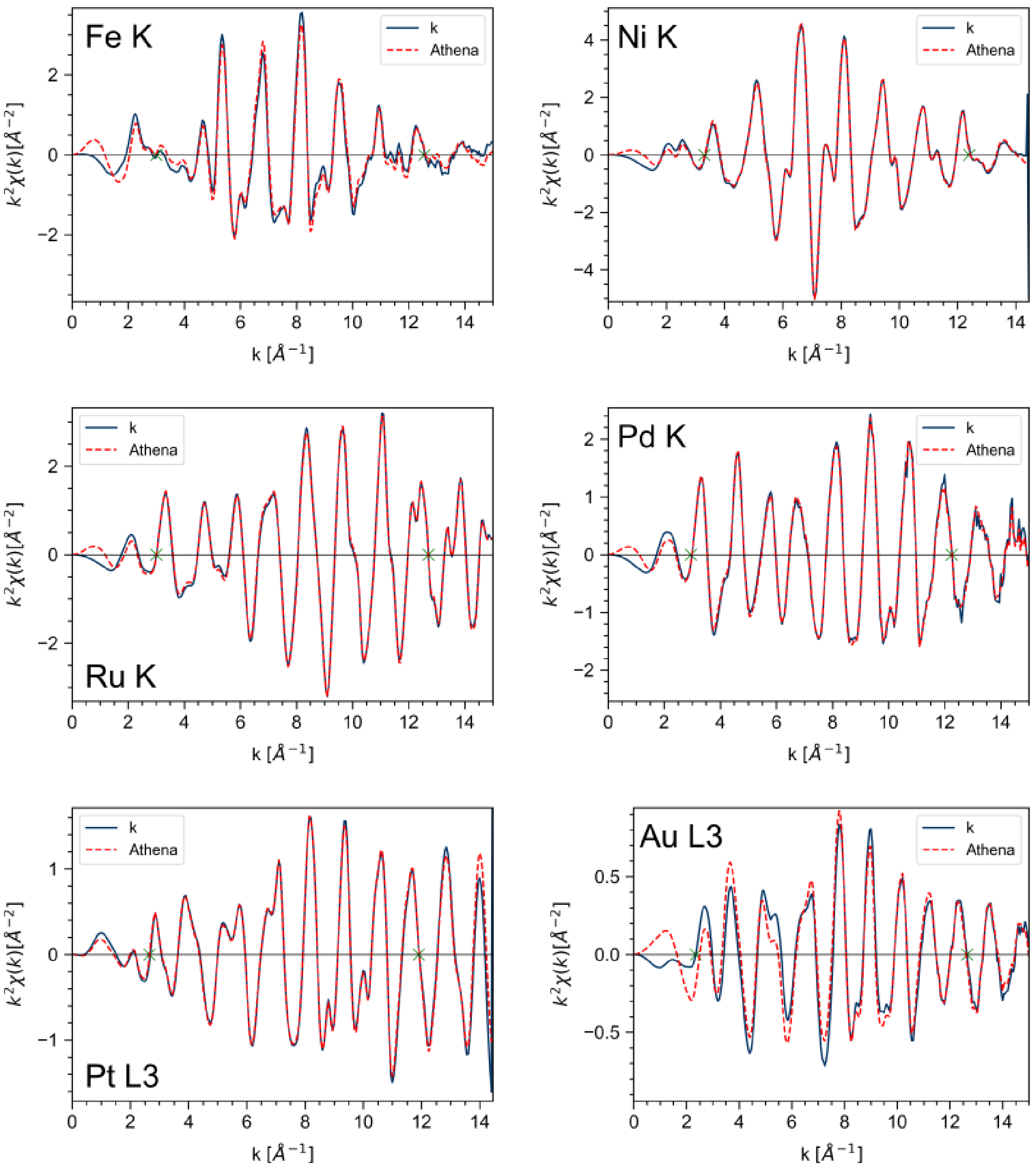

Figure S5 Comparison of Fourier Transformed χ(R) spectra the metal foils in R-space. The red dashed line represents the manually evaluated spectra using the software *Athena* and the blue line the automatically evaluated spectra.

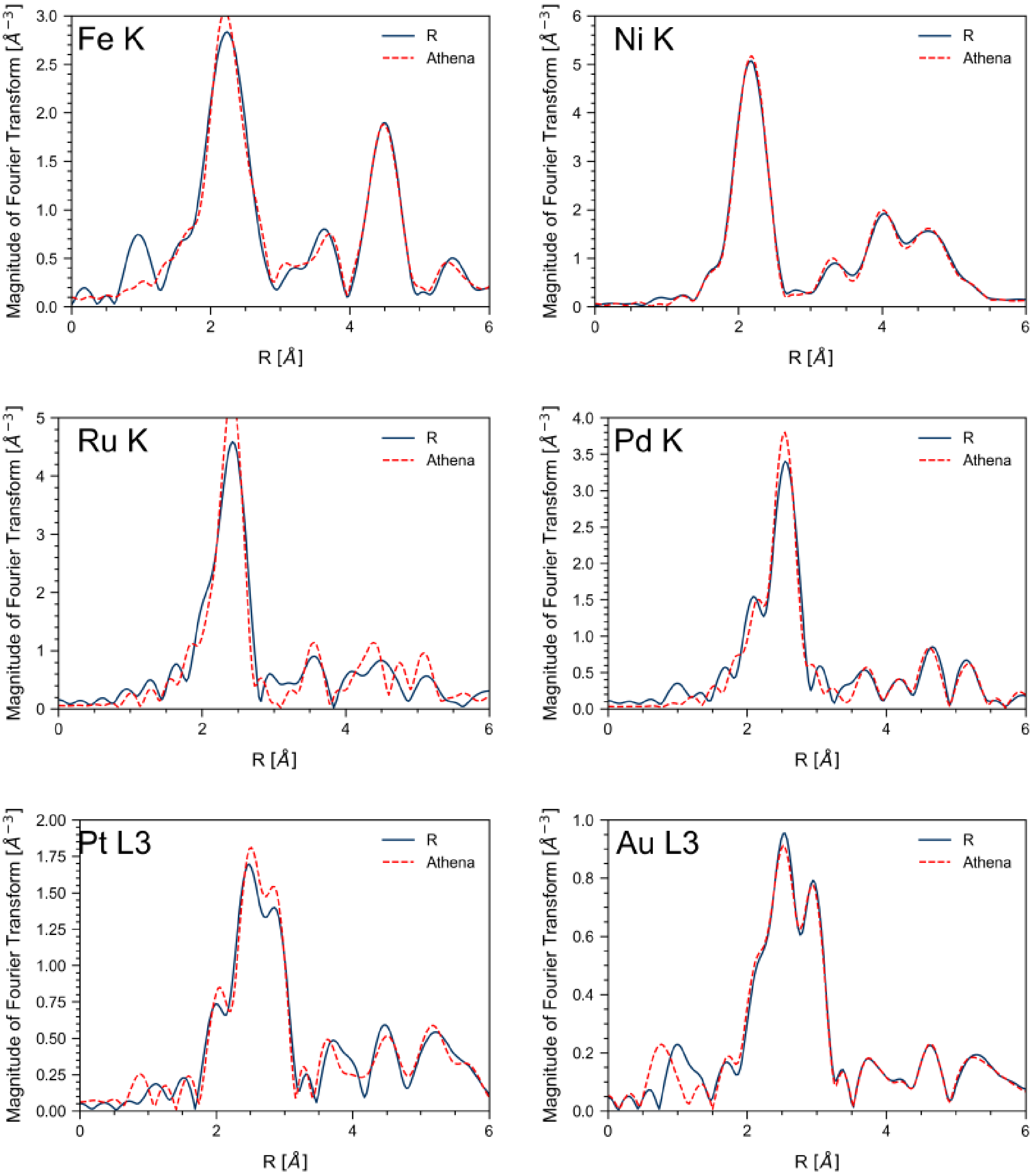

S1. Technical details and implementation

Python3 (Rossum & Drake, 2009) was one of the main languages used in this project, while Django 4.0 (Django. Django Software Foundation, 2023) and the Django REST framework 3.1 (Django REST Framework. Encode OSS, 2023) were used to build the web-server and provide a RESTful API. This includes the development of the front-end using Django-templates, HTML, CSS and JavaScript to provide a user-friendly, modern and responsive interface. Django 4.0 was chosen, because of its robustness, scalability and because it can handle large amount of data. The ASGI (Asynchronous Server Gateway Interface) used to serve the web-server API is Uvicorn (Uvicorn. Encode OSS., 2023), a fast and efficient server that is designed for serving APIs built with Django and Django REST framework.

SciCat (Pithan, 2023) is an open-source software project that provides a comprehensive data management solution for the scientific community and specifically the SciCat backend-next (Pithan, 2022) component. We are currently working with the package version published on November 9th, 2022. In the context of this project, SciCat provided a centralized repository for storing and managing metadata related to any uploaded dataset. The responsible NoSQL database management system, that communicates with the SciCat API is MongoDB (MongoDB. MongoDB, Inc., 2023), where the metadata and graphs generated from the submitted data are stored in a JavaScript Object Notation (JSON)-format (Pezoa, 2016). PostgreSQL (PostgreSQL. PostgreSQL Global Development Group, 2023), an open-source relational database management system, is used to store metadata about the uploaded data-file itself, because of limitations in the SciCat API. PostgreSQL communicates via the Django REST framework with an object-storage API, where every uploaded data-file is stored in a bucket. Because of its accessibility and user-friendliness for an object-storage provider, the first choice here was AWS-S3 (Amazon web services (Amazon S3 storage. Amazon Web Services, 2023)), while other interfaces may also be used. Since any direct communication between the web-server and the SciCat API would have failed, OpenAPI (The OpenAPI specification. The Linux Foundation, 2023), an open-source specification for RESTful APIs, was utilised to generate a client, that was customised, and functions as a bridge between the endpoints of both the SciCat API and the RefXAS API.

Further tools, that were used, are Docker (Merkel, 2014), an open-source tool for automating the deployment, scaling and management of containerized applications such as the RefXAS web-server, and Docker-compose, a tool for defining and running multi-container Docker applications. Docker-compose uses a YAML (YAML. The YAML Project, 2023) file to define all necessary services, that ought to be working together, such as:

- reverse-proxy; a network service that allows incoming requests to be redirected to the regarding back-end service
- MongoDB (persistent)
- PostgreSQL (persistent)
- SciCat, depends on MongoDB
- RefXAS, depends on SciCat and PostgreSQL

All services are started with one single command and are currently hosted on a VM instance as part of the google cloud service (Google Cloud - Virtual Machine Instances. Google Cloud., 2023). Overall, the use of these tools and technologies allowed for the development of a robust, scalable, and efficient reference database, that can be easily used by the community.

S2. Database - and website - design

Upon accessing the website, users are greeted by an adaptive web interface, see Fig. 4, featuring a responsive navigation bar with multiple available options: “Home”, “Upload”, “Search all Datasets (with filters)” and “Team/Contact”. A decentralized Hamburger Navigation displays additionally the “Curator access”, “Add Beamline”, “Added Beamlines” and “Log in/Log out” option and for users with relevant credentials a Login which will give logged in users the option to upload a dataset. A centrally located "Browse all datasets" button is provided for ease of access, and a search bar is positioned in the upper right corner to facilitate quick dataset queries. This allows users to filter datasets based on specific keywords, (also in the metadata) such as “Element x”, “Beamline y” etc. , simplifying the process of locating relevant data. Finally, users will find a hyperlink centrally located to the public page of our database (Paripsa, 2023). A dataset query, for instance, may consist of an element, irrespective of capitalisation, allowing for flexible search capabilities.

After signing into the database portal, the users can upload a dataset. Users need to select the "Upload" option, which navigates them to the "Upload View". In this interface, users can choose the desired dataset and obtain additional information. Upon clicking the "Verify and Upload" button, scripts are executed in the background.

In the context of our web-based Django framework, the *dataset_upload* function is defined within the “views.py”, therefore integrated within our web-server. The function provides an essential bridge between the user’s data submission and the back-end processing, hence processes the data file content, stores the data file temporarily in a folder on the cloud server, extracts relevant metadata and extracts the header of the data file. In our architecture, the dataset handling service includes a feature that visualizes raw data, *i.e.*, it utilizes a component designed for data reading and visualization, transforming raw data into a plot. This plot is then converted into a base64 encoded JPEG image for efficient web display (see Fig. 6). The raw data plot preview facilitates a visual inspection of the dataset, ensuring the data's quality and consistency visually before integrating it into the reference database. The function also allows users to update the energy range. By specifying new minimum and maximum energy values, the dataset is filtered and adjusted accordingly, enabling a more focused analysis. Furthermore, a second method is called, that extracts the entire header from the data file and caches it in a dictionary. In our implementation, key data structures, such as dictionaries, are cached during the execution of Python scripts. This known caching mechanism stores frequently accessed data in a temporary, fast-access storage layer, allowing the script to bypass redundant processing steps and enhance performance.

Figure S6 Illustrated is the extracted data-file title, a customizable raw preview of the XAFS spectrum, along with "e-range min" and "e-range max" fields that provide users to adjust the energy range as required.

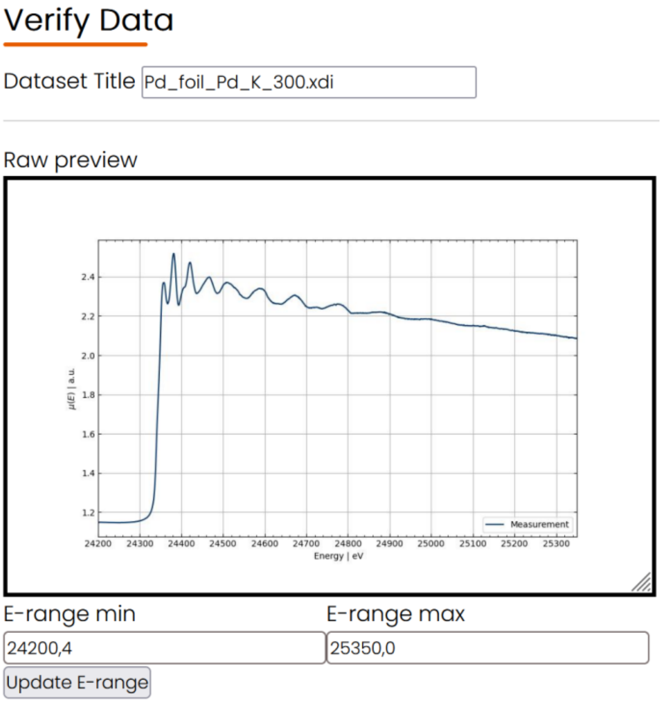

Once the dataset is prepared, users are presented with the automatically extracted title of the data-file, a dynamically generated raw preview of the XAS spectrum upon dataset submission. This preview is encapsulated and can be resized. Additionally, the interface includes “e-range min” and “e-range max” fields, allowing users to specify the energy range of interest. Adjusting these fields triggers a page reload, subsequently displaying the spectrum within the newly defined energy range. Furthermore, the automatically extracted header information is displayed as shown in Fig. 7, left. Here, the full extraction of the data-file header, presented in a size-adjustable textbox, is given. Below this, the user interface (UI) displays most metadata fields as detailed in Table 1. The first section of these metadata fields, labelled as “sample info”, shows six fields that the user is required to fulfil. Additionally, three size-adjustable text boxes are provided for user input on “description”, “general remarks” and “sample preparation”, facilitating a detailed account of the experimental conditions.

In the continued exploration of metadata sections, Figure 7 also presents the "Instrument" section, depicting seven metadata-fields and two drop-down lists. These lists allow users to select the "element" and the "edge" relevant to their measurement. The "edge" list includes options such as "K", "L1", "L2", "L3", and "M4,5", while the "element" list contains all known elements of the periodic table. Notably, the final metadata field, "Max k-range," is specifically tailored to Extended X-ray Absorption Fine Structure (EXAFS) measurements. The subsequent section, "Bibliography", incorporates metadata fields for "DOI", "Reference", and three mandatory fields, framed in red, emphasizing their required completion to prevent a “500 Internal Server Error” response from the SciCat back-end. Finally, the user interface presents the "Data" section, featuring two drop-down lists: "Source," allowing users to specify if the measurement was conducted at a synchrotron or a laboratory facility, and "Measurement mode," to select the type of measurement, such as Absorption, Fluorescence, electron

yield, etc. The interface also provides users with the option to either terminate the entire upload process by selecting the "Cancel" button or finalize it by clicking the "Submit" button.

Figure S7 Left: The data-file header and metadata fields, including a dedicated section for "sample info" with additional text boxes for detailed descriptions and remarks. Right: The metadata sections “Instrument” and “Bibliography”, including twelve fields to be filled, two drop-down lists and three mandatory fields crucial to avoid back-end errors, framed in red. Bottom: the “Data” section of the UI, featuring options to select the measurement "Source" and "Mode", along with "Cancel" and "Submit" buttons to control the upload process.

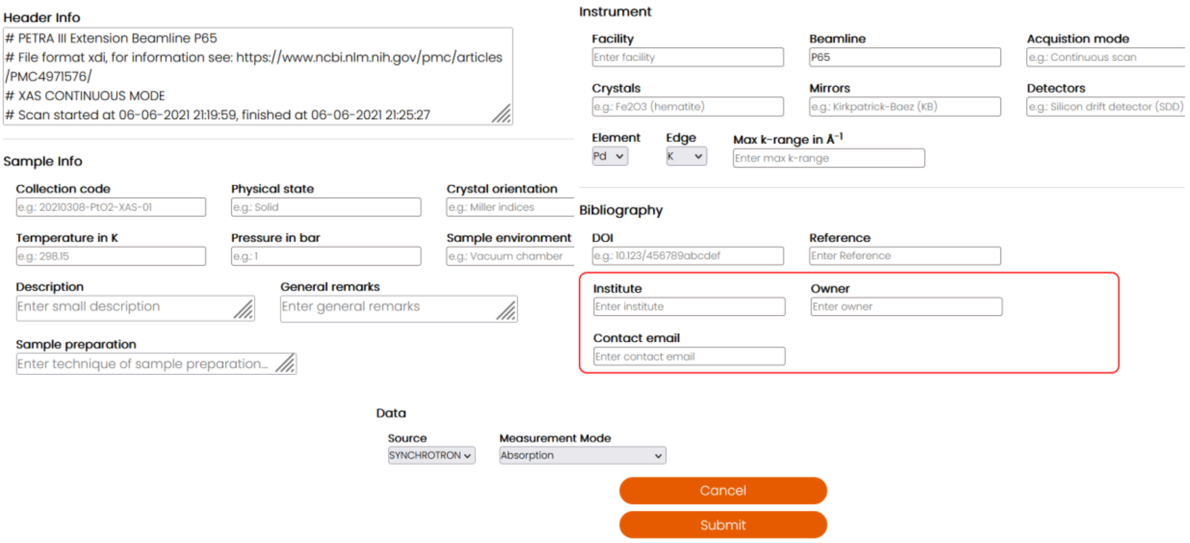

Figure S8 The “Add Beamline” - mask. After choosing “Synchrotron”, the displayed input fields can be filled in by curators / users.

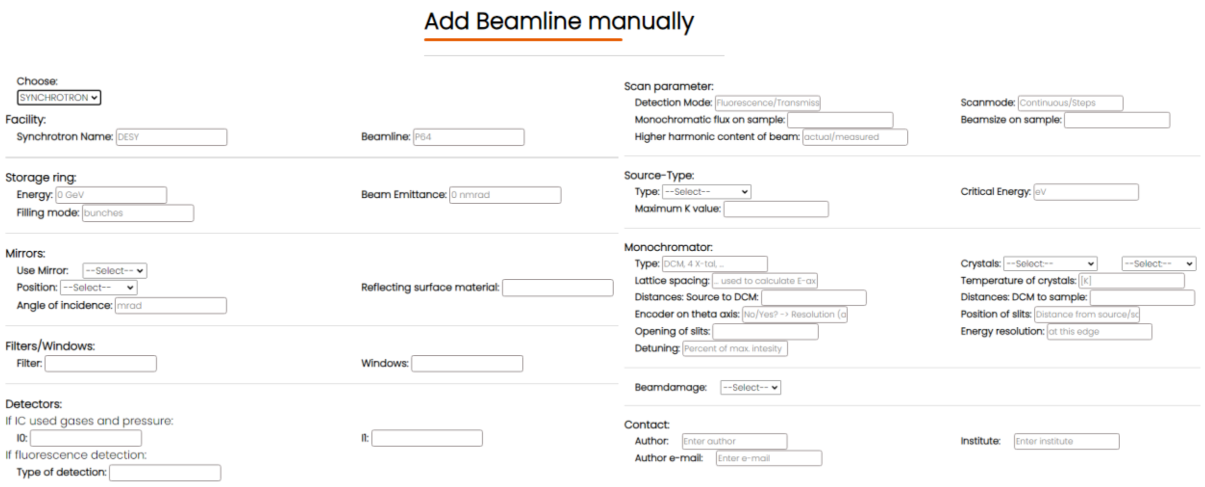

The "Add Beamline" feature on our website, see Fig. 8, offers an intuitive interface for curators / users to input comprehensive details about the experimental set-up at a synchrotron facility. This dedicated mask captures essential data across various categories including facility specifics, storage ring parameters, mirrors, filters, windows, detection modes, and monochromator characteristics. It is designed to

facilitate the precise documentation of experimental conditions, directly contributing to the RefXAS database's robustness and ensuring the reproducibility of results. Please see Table S1 for more details.

We are aiming to enhance each dataset with relevant metadata: when a dataset is uploaded under a specific beamline, it will automatically associate with the corresponding beamline profile already established in the system. This ensures that each dataset inherits a detailed set of information from the beamline's recorded parameters.

Table S4 The table shows the complete benchmarking results.

Total transactions	7,884 hits
Server availability	100%
Total elapsed time	30.04 seconds
Data transferred	132.40 MB
Average response time	0.28 seconds
Transaction rate	262.45 transactions per second
Throughput	4.41 MB/sec
Concurrency level	74.60
Successful transactions	7,132
Failed transactions	0
Longest transaction	5.56 seconds
Shortest transaction	0.06 seconds
